# Supplementary figures and images for: Iodine increases pulmonary type I interferon responses and decreases covid-19 disease severity: Results from an open label randomized clinical trial
Source: PLoS One. 2026 Feb 2;21(2):e0341126. doi: 10.1371/journal.pone.0341126 (PMC12863515; doi:10.1371/journal.pone.0341126)

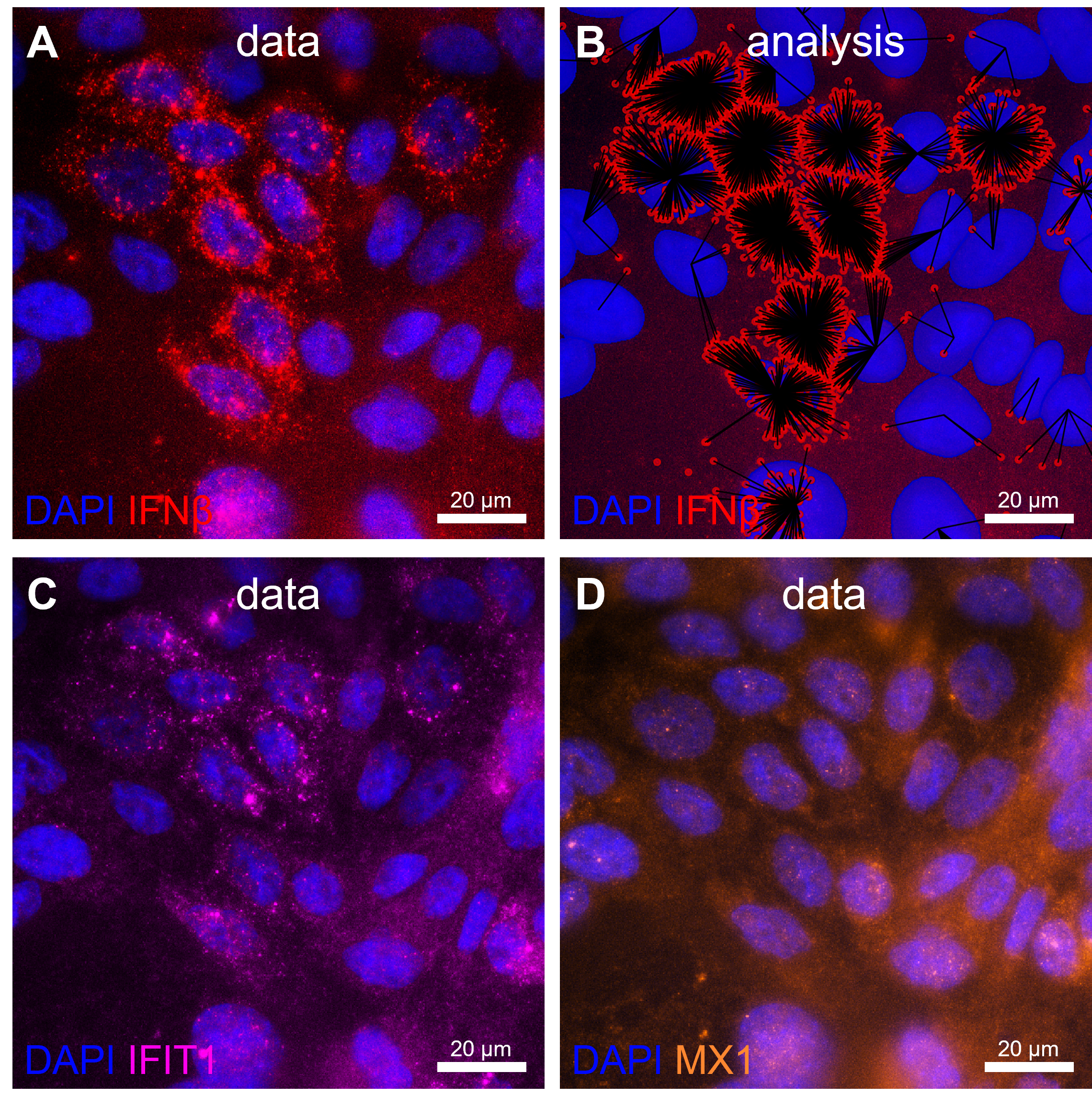

Supplement: S1 Fig — A IFNβ expression in Calu-3 lung epithelium. B automated image analyses of IFNβ spot detection and assignment to nearest nucleus. C IFIT1 expression in Calu-3 lung epithelium. D MX1 expression in Calu-3 lung epithelium. (PNG) [file pone.0341126.s001.png]

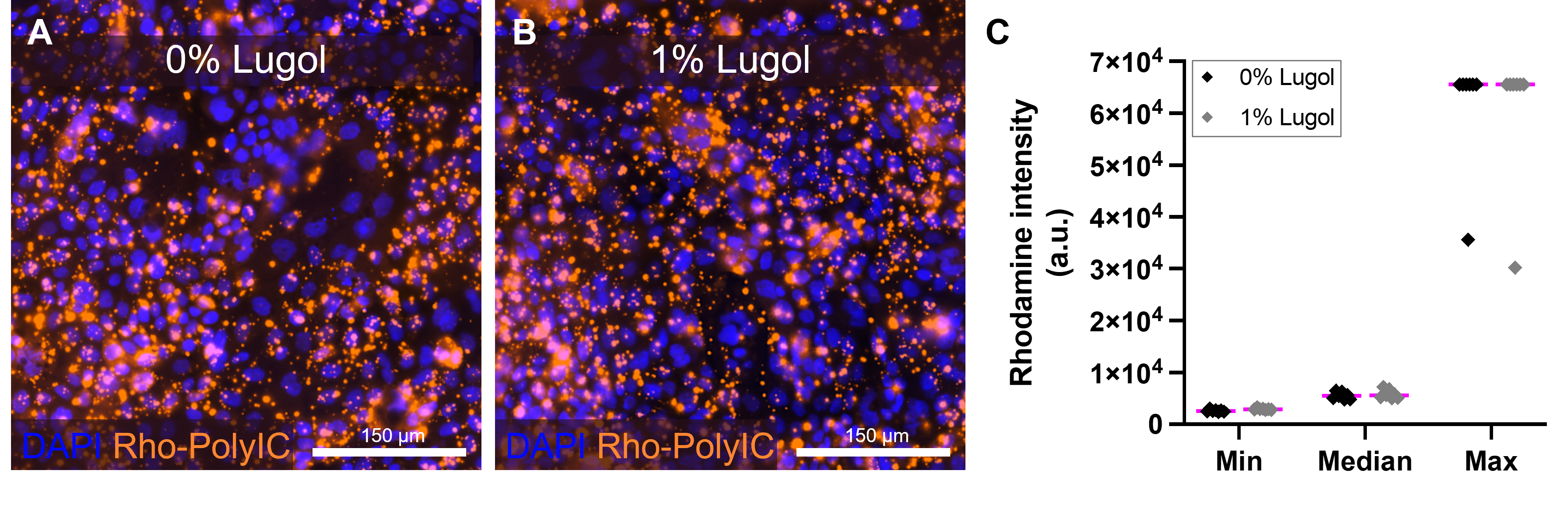

Supplement: S2 Fig — A Representative microscopy image of Calu-3 lung epithelium transfected with rhodamine-labeled PolyIC using lipofectamine for 6 hours. B Representative microscopy image as in A. Cells were treated with 1% Lugol solution for 7 hours in total, starting 1 hour prior transfection with rhodamine-labeled PolyIC for 6 hours. C Quantification of rhodamine signal of 7 representative regions per condition, across n = 2. (PNG) [file pone.0341126.s002.png]

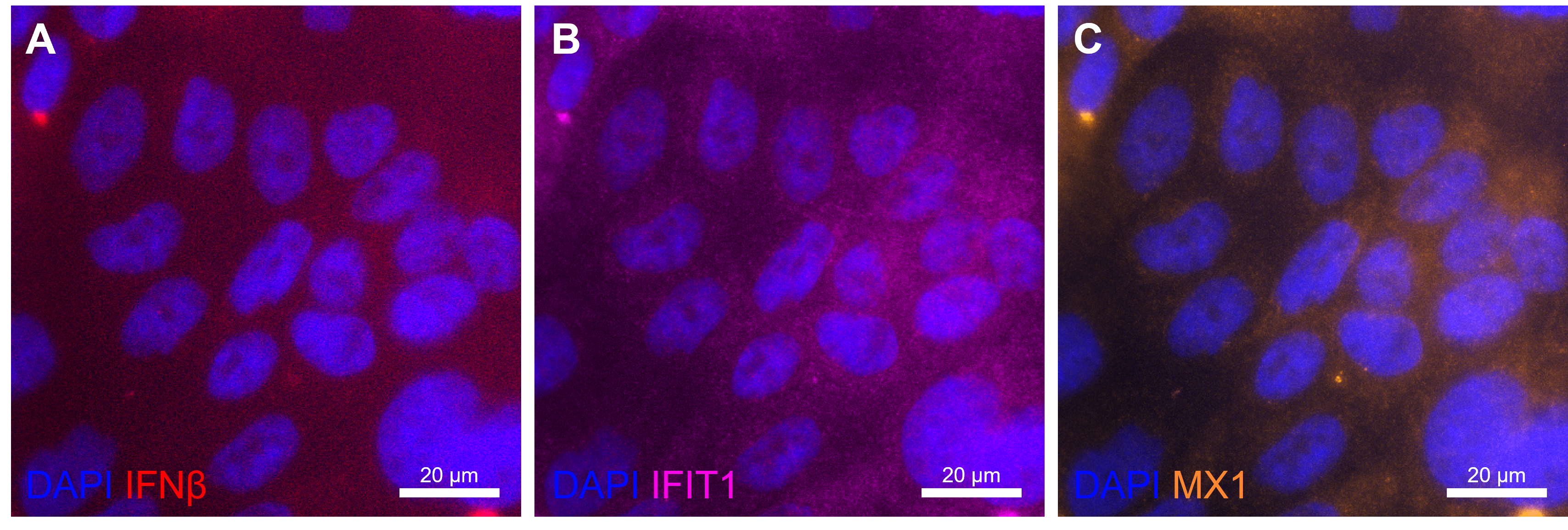

Supplement: S3 Fig — A IFNβ expression, close, if not zero, in Calu-3 lung epithelium. B IFIT1 expression in Calu-3 lung epithelium. C MX1 expression in Calu-3 lung epithelium. (PNG) [file pone.0341126.s003.png]
