# Supplementary material for: Iodine increases pulmonary type I interferon responses and decreases covid-19 disease severity: Results from an open label randomized clinical trial
Source: PLoS One. 2026 Feb 2;21(2):e0341126. doi: 10.1371/journal.pone.0341126 (PMC12863515; doi:10.1371/journal.pone.0341126)
Supplement: S1 File — (PDF) [file pone.0341126.s004.pdf]

RESEARCH PROTOCOL (Translation)

Effect of iodine treatment on COVID-19 infection in clinical patients

PROTOCOL TITLE 'Effect Iodine treatment in patients with COVID-19 infection'

**Protocol ID**	**N20.0490**
**Short title**	**Jodium behandeling Coronavirus**
**EudraCT number**	**2020-001852-16**
**Version**	**3**
**Date**	**20200603**
**Coordinating investigator/project leader**	***R.A.M. Traksel Maxima MC, r.traksel@mmc.nl***
**Principal investigator(s) (in Dutch: hoofdonderzoeker/ uitvoerder)**	***R.A.M. Traksel Maxima MC, r.traksel@mmc.nl***<br>***R.H. Verheesen Maxima MC, rh.verheesen@mmc.nl***<br>***J.C.A. Broen Maxima MC, j.broen@mmc.nl***
**Sponsor (in Dutch: verrichter/opdrachtgever)**	***Maxima MC***
**Subsidising party**	
**Independent expert (s)**	***M.Y. Bongers, Maxima MC, m.bongers@mmc.nl***
**Laboratory sites <*if applicable*>**	***N.A.***
**Pharmacy**	**P.A.G. de Klaver, Maxima MC, *p.deKlaver@mmc.nl***

PROTOCOL SIGNATURE SHEET

Name	Signature	Date
R.A.M. Traksel<br><br>R.H. Verheesen		
J.C.A. Broen		

TABLE OF CONTENTS

1. INTRODUCTION AND RATIONALE........................ **Fout! Bladwijzer niet gedefinieerd.**
2. OBJECTIVES.. **Fout! Bladwijzer niet gedefinieerd.**
3. STUDY DESIGN .... **Fout! Bladwijzer niet gedefinieerd.**
4. STUDY POPULATION.. **Fout! Bladwijzer niet gedefinieerd.**
   4.1 Population (base)... **Fout! Bladwijzer niet gedefinieerd.**
   4.2 Inclusion criteria .... **Fout! Bladwijzer niet gedefinieerd.**
   4.3 Exclusion criteria... **Fout! Bladwijzer niet gedefinieerd.**
   4.4 Sample size calculation .... **Fout! Bladwijzer niet gedefinieerd.**
5. TREATMENT OF SUBJECTS .... **Fout! Bladwijzer niet gedefinieerd.**
   5.1 Investigational product/treatment...................... **Fout! Bladwijzer niet gedefinieerd.**
   5.2 Use of co-intervention (if applicable)................. **Fout! Bladwijzer niet gedefinieerd.**
   5.3 Escape medication (if applicable) .... **Fout! Bladwijzer niet gedefinieerd.**
6. INVESTIGATIONAL PRODUCT .... **Fout! Bladwijzer niet gedefinieerd.**
   6.1 Name and description of investigational product(s)**Fout! Bladwijzer niet gedefinieerd.**
   6.2 Summary of findings from non-clinical studies.. **Fout! Bladwijzer niet gedefinieerd.**
   6.3 Summary of findings from clinical studies......... **Fout! Bladwijzer niet gedefinieerd.**
   6.4 Summary of known and potential risks and benefits**Fout! Bladwijzer niet gedefinieerd.**
   6.5 Description and justification of route of administration and dosage**Fout! Bladwijzer niet gedefinieerd.**
   6.6 Dosages, dosage modifications and method of administration**Fout! Bladwijzer niet gedefinieerd.**
   6.7 Preparation and labelling of Investigational Medicinal Product**Fout! Bladwijzer niet gedefinieerd.**
   6.8 Drug accountability .... **Fout! Bladwijzer niet gedefinieerd.**
7. NON-INVESTIGATIONAL PRODUCT .... **Fout! Bladwijzer niet gedefinieerd.**
   7.1 Name and description of non-investigational product(s)**Fout! Bladwijzer niet gedefinieerd.**
   7.2 Summary of findings from non-clinical studies.. **Fout! Bladwijzer niet gedefinieerd.**
   7.3 Summary of findings from clinical studies......... **Fout! Bladwijzer niet gedefinieerd.**
   7.4 Summary of known and potential risks and benefits**Fout! Bladwijzer niet gedefinieerd.**
   7.5 Description and justification of route of administration and dosage**Fout! Bladwijzer niet gedefinieerd.**
   7.6 Dosages, dosage modifications and method of administration**Fout! Bladwijzer niet gedefinieerd.**
   7.7 Preparation and labelling of Non Investigational Medicinal Product**Fout! Bladwijzer niet gedefinieerd.**
   7.8 Drug accountability .... **Fout! Bladwijzer niet gedefinieerd.**
8. METHODS .... **Fout! Bladwijzer niet gedefinieerd.**

8.1 Study parameters/endpoints .... Fout! Bladwijzer niet gedefinieerd.
8.1.1 Main study parameter/endpoint .... Fout! Bladwijzer niet gedefinieerd.
8.1.2 Secondary study parameters/endpoints (if applicable)Fout! Bladwijzer niet gedefinieerd.
8.1.3 Other study parameters (if applicable)....... Fout! Bladwijzer niet gedefinieerd.
8.2 Randomisation, blinding and treatment allocationFout! Bladwijzer niet gedefinieerd.
8.3 Study procedures .... Fout! Bladwijzer niet gedefinieerd.
8.4 Withdrawal of individual subjects .... Fout! Bladwijzer niet gedefinieerd.
8.4.1 Specific criteria for withdrawal (if applicable)Fout! Bladwijzer niet gedefinieerd.
8.5 Replacement of individual subjects after withdrawalFout! Bladwijzer niet gedefinieerd.
8.6 Follow-up of subjects withdrawn from treatmentFout! Bladwijzer niet gedefinieerd.
8.7 Premature termination of the study.................. Fout! Bladwijzer niet gedefinieerd.
9. SAFETY REPORTING... Fout! Bladwijzer niet gedefinieerd.
9.1 Temporary halt for reasons of subject safety ……………………………………….Fout! Bladwijzer niet gedefinieerd.
9.2 AEs, SAEs and SUSARs .... Fout! Bladwijzer niet gedefinieerd.
9.2.1 Adverse events (AEs) .... Fout! Bladwijzer niet gedefinieerd.
9.2.2 Serious adverse events (SAEs)................ Fout! Bladwijzer niet gedefinieerd.
9.2.3 Suspected unexpected serious adverse reactions (SUSARs)Fout! Bladwijzer niet gedefinieerd.
9.3 Annual safety report... Fout! Bladwijzer niet gedefinieerd.
9.4 Follow-up of adverse events .... Fout! Bladwijzer niet gedefinieerd.
9.5 [Data Safety Monitoring Board (DSMB) / Safety Committee]Fout! Bladwijzer niet gedefinieerd.
10. STATISTICAL ANALYSIS .... Fout! Bladwijzer niet gedefinieerd.
10.1 Primary study parameter(s) .... Fout! Bladwijzer niet gedefinieerd.
10.2 Secondary study parameter(s).......................... Fout! Bladwijzer niet gedefinieerd.
10.3 Other study parameters .... Fout! Bladwijzer niet gedefinieerd.
10.4 Interim analysis (if applicable).......................... Fout! Bladwijzer niet gedefinieerd.
11. ETHICAL CONSIDERATIONS .... Fout! Bladwijzer niet gedefinieerd.
11.1 Regulation statement....................................... Fout! Bladwijzer niet gedefinieerd.
11.2 Recruitment and consent .... Fout! Bladwijzer niet gedefinieerd.
11.3 Objection by minors or incapacitated subjects (if applicable)Fout! Bladwijzer niet gedefinieerd.
11.4 Benefits and risks assessment, group relatednessFout! Bladwijzer niet gedefinieerd.
11.5 Compensation for injury .... Fout! Bladwijzer niet gedefinieerd.
11.6 Incentives (if applicable) .... Fout! Bladwijzer niet gedefinieerd.
12. ADMINISTRATIVE ASPECTS, MONITORING AND PUBLICATIONFout! Bladwijzer niet gedefinieerd.

12.1 Handling and storage of data and documents .. **Fout! Bladwijzer niet gedefinieerd.**
12.2 Monitoring and Quality Assurance .... **Fout! Bladwijzer niet gedefinieerd.**
12.3 Amendments .... **Fout! Bladwijzer niet gedefinieerd.**
12.4 Annual progress report .... **Fout! Bladwijzer niet gedefinieerd.**
12.5 End of study report .... **Fout! Bladwijzer niet gedefinieerd.**
12.6 Public disclosure and publication policy .... **Fout! Bladwijzer niet gedefinieerd.**
13. STRUCTURED RISK ANALYSIS .... **Fout! Bladwijzer niet gedefinieerd.**
13.1 Potential issues of concern .... **Fout! Bladwijzer niet gedefinieerd.**
13.2 Synthesis .... **Fout! Bladwijzer niet gedefinieerd.**
14. REFERENCES 25

LIST OF ABBREVIATIONS AND RELEVANT DEFINITIONS

**ABR**	**General Assessment and Registration form (ABR form), the application form that is required for submission to the accredited Ethics Committee; in Dutch: Algemeen Beoordelings- en Registratieformulier (ABR-formulier)**
**AE**	**Adverse Event**
**AR**	**Adverse Reaction**
**CA**	**Competent Authority**
**CCMO**	**Central Committee on Research Involving Human Subjects; in Dutch: Centrale Commissie Mensgebonden Onderzoek**
**CV**	**Curriculum Vitae**
**DSMB**	**Data Safety Monitoring Board**
**EU**	**European Union**
**EudraCT**	**European drug regulatory affairs Clinical Trials**
**GCP**	**Good Clinical Practice**
**GDPR**	**General Data Protection Regulation; in Dutch: Algemene Verordening Gegevensbescherming (AVG)**
**IB**	**Investigator’s Brochure**
**IC**	**Informed Consent**

**IMP**	**Investigational Medicinal Product**
**IMPD**	**Investigational Medicinal Product Dossier**
**METC**	**Medical research ethics committee (MREC); in Dutch: medisch-ethische toetsingscommissie (METC)**
**(S)AE**	**(Serious) Adverse Event**
**SPC**	**Summary of Product Characteristics; in Dutch: officiële productinformatie IB1-tekst**
**Sponsor**	**The sponsor is the party that commissions the organisation or performance of the research, for example a pharmaceutical company, academic hospital, scientific organisation or investigator. A party that provides funding for a study but does not commission it is not regarded as the sponsor, but referred to as a subsidising party.**
**SUSAR**	**Suspected Unexpected Serious Adverse Reaction**
**UAVG**	**Dutch Act on Implementation of the General Data Protection Regulation; in Dutch: Uitvoeringswet AVG**
**WMO**	**Medical Research Involving Human Subjects Act; in Dutch: Wet Medisch-wetenschappelijk Onderzoek met Mensen**

SUMMARY

Rationale: Iodine has germicidal properties and contributes to the defense mechanism against pathogens. In addition, iodine has anti-inflammatory and immune-modulating properties that may have a beneficial effect in the treatment of COVID-19 infections. This leads to our hypothesis that iodine is effective in the treatment of COVID-19 disease.

Objective: To demonstrate that iodine treatment has a beneficial effect on the course of COVID-19 disease. Beneficial effect is defined as a reduced risk of deterioration, such as transfer from a regular ward to the ICU, death in the regular ward, or death in the ICU.

Study design: Open-label randomized trial with a control group.

Study population: All patients aged 18 years and older who are admitted to the hospital and test positive for SARS-CoV-2 infection will be recruited for the study. Patients must be mentally competent and able to read the patient information sheet and sign the informed consent form.

Intervention: Patients in the intervention group will receive, for 8 consecutive days, once daily, ¼ Potassium Iodide 65 mg tablet. The control group will receive no supplement and no placebo.

Main study parameters/endpoints:

Primary endpoint: clinically relevant deterioration. Deterioration is defined as:

1. From baseline on regular ward to ICU
2. From baseline on regular ward and death
3. From baseline in ICU and death

Secondary endpoint: total number of days of hospital admission and total number of deaths.

Nature and extent of the burden and risks associated with participation, benefit and group relatedness: When a patient is randomized and placed in the treatment group, a beneficial effect on the course of the COVID-19 infection is to be expected. The patient must then take ¼ Potassium Iodide 65 mg tablet once daily for 8 consecutive days. The risk, given the exclusion criteria, is very limited and in principle temporary.

1. INTRODUCTION AND RATIONALE

Iodine has a germicidal effect and contributes to the defense mechanism against pathogens [1]. The SARS-CoV-2 virus has, in addition to a protective protein shell, the capsid, an outer lipid membrane—the envelope—in which proteins are also anchored. Hemagglutinin (HA) is a surface protein that enables recognition of host cells and causes “injection” of the viral genome by inducing fusion of the endosomal membranes. Neuraminidase (NA) is another surface protein that ensures that virus particles, after replication in cells, can detach from these cells and spread further. It does this by cleaving sialic acid residues. Iodine reduces the virulence of viruses through interaction with various surface proteins. This leads to degeneration of the capsid and thereby to virus inactivation [14]. Additionally, iodine inhibits HA and NA activity, making it more difficult for the virus to enter the host cell and to spread outside the cell [16,17,21]. Iodine may also cause destabilization of the envelope through a reaction with unsaturated carbon compounds [15]. Iodine has antiviral properties against coronaviruses [22,23]. Recently, a chemical interaction has been described between iodine and the Coronavirus spike (S) protein, which facilitates fusion of the virus with the host cell, and with the ACE2 receptors. These findings strongly suggest a beneficial effect of iodine treatment in Coronavirus infections [27,28].

COVID-19 disease can be fatal as a result of an overreaction of the body’s immune system, called a cytokine storm. More pro-inflammatory cytokines are then produced, such as IL-2, IL-8, IFN-gamma, and TNF-alpha. Treatment with iodine at non-toxic dosages leads to a reduction of these cytokines [2,18,19]. Additionally, an excess of Reactive Oxygen Species (ROS, radicals) is produced, which are toxic to pathogens but at the same time can also be harmful to the body itself [20]. Neutralizing this excess of radicals with antioxidants can have a beneficial effect on the course of conditions associated with high oxidative stress [20]. Iodine is a strong antioxidant and neutralizes excess ROS [3,24].

Iodine is secreted via the mucous membranes in the mouth and trachea, partly via Sodium-Iodide symporters. Furthermore, there is a relationship between iodine status and the concentration of iodine in saliva [13,25]. Additionally, iodine has anti-inflammatory and immune-modulating properties that may have a beneficial effect in the treatment of COVID-19 infections [2,3]. In multiple case reports, a beneficial effect of iodine treatment in viral infections is described [4,5].

In epidemiological research into the number of deceased COVID-19 patients, a striking difference is found between Japan and Italy. On April 1, 2020, Japan had 57 deaths with 126 million inhabitants (0.5/million), and Italy had 12,428 deaths with 60 million inhabitants (207/million) [6]. Furthermore, Japanese people are known for their high iodine intake through food, in contrast to Italians who are iodine deficient. The iodine status, measured in Urinary Iodine Concentration (UIC) according to WHO criteria, is 282 mcg/L in Japan and 84 mcg/L in Italy [7,8,9]. These two findings suggest a protective effect of iodine against COVID-19 infections. This leads to our hypothesis that iodine is effective in the treatment of COVID-19 infections. To test the hypothesis, we want to conduct research into the treatment with iodine in COVID-19 infected patients.

2. OBJECTIVES

Primary endpoint: Clinically relevant deterioration. Deterioration is defined as:

- From baseline on general ward to ICU
- From baseline on general ward and death
- From baseline in ICU and death

Secondary endpoint:

- Total number of days of hospital admission
- Total number of deaths

3. STUDY DESIGN

Open-label randomized clinical trial with control group.

4. STUDY POPULATION

4.1 Population

Hospitalized patients with COVID-19 disease in Maxima MC.

4.2 Inclusion criteria

All patients aged 18 years and older who test positive for SARS-CoV-2 infection may participate in this study.

4.3 Exclusion criteria

Thyroid disorder or treatment such as goiter, thyroidectomy, radioactive iodine, medications related to thyroid dysfunction or Amiodarone. The investigator must have access to the medical history and medication use of the subject. If necessary, contact will be made with the general practitioner or other hospitals to obtain the required information. If no or insufficient data can be obtained, the subject cannot participate in the study.

4.4 Sample size calculation

If a less optimistic scenario is assumed, with 25% deterioration (transfer to ICU or death on ward or in ICU) in the control arm and 10% in the intervention group, the power calculation is as follows: 25% vs 10% → 100 subjects per group → 200 subjects total → 10% dropout → 224 subjects needed An interim analysis is planned with stopping rule according to O'Brien Fleming, for which no adjustment of the sample size is needed. After 50 patients in each arm

5. TREATMENT OF SUBJECTS

For 8 consecutive days, once daily ¼ Potassium Iodide 65 mg tablet. Total iodine dosage during the treatment course amounts to 130 mg. After 8 days, a significant reduction in SARS-CoV-2 virus titers in nasal and throat swabs is observed [26].

5.1 Investigational product/treatment

Potassium Iodide 65 mg tablet

5.2 Use of co-intervention (if applicable)

Patients receive the same standard treatment as other patients with a COVID-19 infection. There are no restrictions on the use of other medication.

5.3 Escape medication (if applicable)

Not applicable

6. INVESTIGATIONAL PRODUCT

6.1 Name and description of investigational product(s)

Potassium Iodide 65 mg tablet

6.2 Summary of findings from non-clinical studies

No known studies

6.3 Summary of findings from clinical studies

No known studies

6.4 Summary of known and potential risks and benefits

The likelihood of side effects is derived from the listings in the pharmacotherapeutic compass for Potassium Iodide 65 mg tablets. The side effects listed there are relatively mild and in principle transient. In rare cases, in patients with pre-existing thyroid disorders, thyrotoxicosis may occur. Temporary medicinal treatment may then be necessary to correct thyroid dysfunction. However, this is not expected since thyroid disorders or medication related to thyroid dysfunction are exclusion criteria. Previous studies show that administering relatively high doses of iodine is safe [10,11,12].

6.5 Description and justification of route of administration and dosage

Oral administration for 8 days, once daily ¼ Potassium Iodide 65 mg tablet is minimally burdensome for the patient and easy to administer by nursing staff. Furthermore, the tablets are very inexpensive (€0.01 per tablet according to the pharmacotherapeutic compass). Various in vitro studies and animal model research have identified different required concentrations to achieve virus inactivation. These studies focused particularly on viruses with an envelope, as the SARS-CoV-2 virus also has one. Required concentrations for envelope virus inactivation range between 10 μM and 80 μM iodine [2, 3, 7, 21, 22].

To estimate the required dosage, the following assumptions were made:

- The iodine concentration in alveolar fluid is 20 times higher than in serum [2]. This concentration difference can be achieved through active transport of iodine to the lumen via Sodium-Iodide Symporters in the mucosa.
- For an average weight of 80 kg, the amount of body water is approximately 50 liters.
- Of the administered daily ¼ Potassium Iodide 65 mg tablet, 100% (16.5 mg) is absorbed.
- The half-life of iodine measured in alveolar lung fluid after a single Potassium Iodide administration in animal models is approximately 24 hours [2]. After a single intake of Potassium Iodide, the iodine level in serum returns to baseline within 24 to 48 hours [24].
- Molar mass of Potassium is 40 g and molar mass of Iodine is 127 g, totaling 167 g. Therefore, in 16.5 mg Potassium Iodide, there is (127/167) × 16.5 mg = 12.5 mg Iodine. This corresponds to (0.0125/127) = 98 μmol iodine.
- With a volume of 50 liters of body water, the concentration of iodine is 98/50 = 2 μM
- Of the total amount of iodine in the body, half (6 mg to 14 mg) is stored in the thyroid [23]. The other half is assumed to be peripherally available. So in the body itself, 6 to 14 mg iodine is available in 50 liters of body water. This corresponds to 6/50 = 0.12 mg (0.94 μmol) and 14/50 = 0.28 mg (2.2 μmol) per liter.
- The concentration calculation in serum is then the sum of existing iodine in serum + the added iodine: 0.94 + 2 = 2.94 μM and 2.2 + 2 = 4.2 μM.
- The iodine concentration in alveolar fluid is 20 times higher than in serum. So the calculated extremes of iodine concentration in serum of 2.94 μM and 4.2 μM correspond to 2.94 × 20 = 58.8 μM and 4.2 × 20 = 84 μM in alveolar lung fluid.
- Literature indicates that the required dosages for inactivation of envelope viruses lie between 10 μM and 80 μM.
- As our calculation shows, our daily dosage of ¼ Potassium Iodide 65 mg tablet falls within the range of required iodine concentrations for inactivation of envelope viruses at alveolar lung fluid level.
- The iodine administration lasts 8 days because a clear reduction in SARS-CoV-2 virus titers is only seen after a week [25]. Additionally, we administer iodine not only for its antiviral properties but also for its antioxidant effect, which is needed to address the free radicals generated during a possible cytokine storm. Therefore, we have chosen a treatment duration of 8 days. The total dosage of the course then amounts to 130 mg Potassium Iodide.

6.6 Dosages, dosage modifications and method of administration

For 8 consecutive days, once daily ¼ Potassium Iodide 65 mg tablet. Distributions are registered by the nurse in the electronic prescribing system of the hospital's EHR.

6.7 Preparation and labelling of Investigational Medicinal Product

This concerns an available medicinal product that will be provided via the hospital pharmacy.

6.8 Drug accountability

Potassium Iodide will only be provided during the hospital stay. The prescription will be recorded in the electronic prescribing system of the hospital's EHR.

7. NON-INVESTIGATIONAL PRODUCT

7.1 Name and description of non-investigational product(s)

Not applicable

7.2 Summary of findings from non-clinical studies

Not applicable

7.3 Summary of findings from clinical studies

Not applicable

7.4 Summary of known and potential risks and benefits

Not applicable

7.5 Description and justification of route of administration and dosage

Not applicable

7.6 Dosages, dosage modifications and method of administration

Not applicable

7.7 Preparation and labelling of Non-Investigational Medicinal Product

Not applicable

7.8 Drug accountability

Not applicable

8. METHODS

8.1 Study parameters/endpoints

8.1.1 Main study parameter/endpoint

Primary endpoint: clinically relevant deterioration. Deterioration is defined as:

- From baseline on general ward to ICU
- From baseline on general ward and death
- From baseline in ICU and death

8.1.2 Secondary study parameters/endpoints (if applicable)

Secondary endpoint:

- Total number of deaths
- Total number of days of hospital admission

If the subject has been admitted to the intensive care unit for part of a calendar day, this day counts as one full day of ICU admission. If the subject has been admitted partly to the ICU and partly to a general ward on a calendar day, this day counts as one full day of ICU admission. If the patient has been admitted to a general ward for part of a calendar day and has died or been discharged on that day, this day counts as one full day of general ward admission.

8.1.3 Other study parameters (if applicable)

BSE, CRP, leukocytes, ferritin, oxygen saturation at admission, and temperature progression. These measurements and tests are performed as part of routine care.

8.2 Randomisation, blinding and treatment allocation

Randomisation via Research Manager

8.3 Study procedures

Review patient information form and sign informed consent. Inclusion period runs from July 1, 2020 to July 1, 2022.

8.4 Withdrawal of individual subjects

Subjects may withdraw from the study at any time and for any reason, without consequences. The investigator may decide to withdraw a subject from the study for urgent medical reasons.

8.4.1 Specific criteria for withdrawal (if applicable)

Not applicable

8.5 Replacement of individual subjects after withdrawal

Dropout has been accounted for in the power calculation. No replacement will take place.

8.6 Follow-up of subjects withdrawn from treatment

Follow-up lasts as long as the patient is hospitalized.

8.7 Premature termination of the study

Unexpected serious side effects may be a reason to stop the study. Administration of Potassium Iodide tablets will then be discontinued immediately.

9. SAFETY REPORTING

9.1 Temporary halt for reasons of subject safety

In accordance with section 10, subsection 4, of the WMO, the sponsor will suspend the study if there is sufficient reason to believe that continuation of the study will jeopardize the health or safety of subjects. The sponsor will notify the accredited METC without undue delay of a temporary halt, including the reason for such action. The study will be suspended pending a further positive decision by the accredited METC. The investigator will ensure that all subjects are kept informed.

9.2 AEs, SAEs and SUSARs

9.2.1 Adverse events (AEs)

Adverse events are defined as any undesirable experience occurring to a subject during the study, whether or not considered related to the investigational product, trial procedure, or experimental intervention. All adverse events reported spontaneously by the subject or observed by the investigator or their staff will be recorded. This information will be stored in the subject’s electronic patient record (EPR).

9.2.2 Serious adverse events (SAEs)

A serious adverse event is any untoward medical occurrence or effect that:

- results in death;
- is life-threatening (at the time of the event);
- requires hospitalization or prolongation of existing hospitalization;
- results in persistent or significant disability or incapacity;
- is a congenital anomaly or birth defect; or
- any other important medical event that did not result in any of the outcomes listed above due to medical or surgical intervention but could have been based on appropriate judgment by the investigator.

An elective hospital admission will not be considered a serious adverse event.

The investigator will report all SAEs to the sponsor without undue delay after obtaining knowledge of the events, except for the following SAEs: not applicable.

The sponsor will report the SAEs through the web portal ToetsingOnline to the accredited METC that approved the protocol, within 7 days of first knowledge for SAEs that result in death or are life-threatening, followed by a maximum of 8 days to complete the initial preliminary report. All other SAEs will be reported within a maximum of 15 days after the sponsor has first knowledge of the serious adverse events.

9.2.3 Suspected unexpected serious adverse reactions (SUSARs)

In addition to the expedited reporting of SUSARs, the sponsor will submit, once a year throughout the clinical trial, a safety report to the accredited METC, competent authority, and competent authorities of the concerned Member States.

This safety report consists of:

- a list of all suspected (unexpected or expected) serious adverse reactions, along with an aggregated summary table of all reported serious adverse reactions, ordered by organ system, per study;
- a report concerning the safety of the subjects, consisting of a complete safety analysis and an evaluation of the balance between the efficacy and the harmfulness of the medicine under investigation.

9.3 Annual safety report

See above under SUSARs.

9.4 Follow-up of adverse events

All AEs will be followed until they have abated or until a stable situation has been reached. Depending on the event, follow-up may require additional tests or medical procedures as indicated, and/or referral to the general physician or a medical specialist.

SAEs need to be reported until the end of the study within the Netherlands, as defined in the protocol.

9.5 Data Safety Monitoring Board (DSMB) / Safety Committee

Not applicable

10. STATISTICAL ANALYSIS

The primary analysis of the endpoint “clinically relevant deterioration” is a Kaplan-Meier survival analysis with the duration being the number of days since study start. Study start is the calendar day on which the first dose of Potassium Iodide tablet is administered. The difference in transfer from general ward to ICU, death on general ward, or death in ICU will be tested using a log-rank test. Hazard ratios with 95% confidence intervals will also be calculated using Cox regression analysis.

Secondary analysis will be performed for death alone and total number of days of hospital admission. For this, a Kaplan-Meier with log-rank test and Cox regression analysis will be performed.

10.1 Primary study parameter(s)

Clinically relevant deterioration expressed as the number of patients transferred from a general ward to the ICU and the number of patients who die on the general ward or in the ICU.

10.2 Secondary study parameter(s)

Total number of deaths and total number of days of hospital admission.

10.3 Other study parameters

BSE, CRP, leukocytes, ferritin, oxygen saturation at admission, and temperature progression. These measurements and tests are performed as part of routine care.

10.4 Interim analysis (if applicable)

Halfway through the study, with 50 inclusions per arm, an interim analysis will be performed. There are two reasons why the study may be stopped early:

1. Due to futility. The study will be stopped if, upon recalculation of the sample size, more than 100 participants per arm are needed or the difference between control and intervention arm is not considered clinically relevant (i.e., less than 5% difference).
2. Due to effectiveness (O'Brien Fleming stopping rule). The study will be stopped at a p-value < 0.0054 for the null hypothesis (i.e., no difference between intervention and reference).

11. ETHICAL CONSIDERATIONS

11.1 Regulation statement

The study will be conducted according to the principles of the Declaration of Helsinki (version, date, see for the most recent version: www.wma.net) and in accordance with the Medical Research Involving Human Subjects Act (WMO) and other guidelines, regulations, and Acts.

11.2 Recruitment and consent

Patients will be recruited by nursing staff, physician assistant in training, or attending physician. Consent will be requested by the investigator.

11.3 Objection by minors or incapacitated subjects (if applicable)

Patients who wish to participate must be 18 years or older and mentally competent.

11.4 Benefits and risks assessment, group relatedness

The likelihood of side effects is derived from the listings in the pharmacotherapeutic compass for Potassium Iodide 65 mg tablets. The side effects listed there are relatively mild and in principle transient. In rare cases, in patients with pre-existing thyroid disorders, thyrotoxicosis may occur. Temporary medicinal treatment may then be necessary to

correct thyroid dysfunction. However, this is not expected since thyroid disorders or medication related to thyroid dysfunction are exclusion criteria. The risk of side effects at the dosage of Potassium Iodide used in this study is very limited. By applying exclusion criteria for thyroid disorders and medication related to thyroid dysfunction, the chance of thyrotoxicosis is virtually eliminated. Other side effects are relatively minor and usually transient. There are no long-term health risks expected with this short-term Potassium Iodide treatment. The potential benefits for the patient may be significant, such as a better chance of survival, faster recovery, and shorter duration of hospital admission. Furthermore, there may be less irreversible lung damage, so that the quality of life after the illness period will be better.

11.5 Compensation for injury

Subject insurance has been applied for.

11.6 Incentives (if applicable)

Not applicable

12. ADMINISTRATIVE ASPECTS, MONITORING AND PUBLICATION

12.1 Handling and storage of data and documents

Research Manager is used in a secure hospital environment to store data. Source data is stored in a secured Excel file.

12.2 Monitoring and Quality Assurance

Given the short duration of the study, practical arrangements will be made to organize this remotely. Contact has been made with CTCM and an initial visit took place by telephone on April 16.

12.3 Amendments

Amendments are changes made to the research after a favorable opinion by the accredited METC has been given. All amendments will be notified to the METC that gave a favorable opinion. Non-substantial amendments will not be notified to the accredited METC and the competent authority, but will be recorded and filed by the sponsor.

12.4 Annual progress report

The sponsor/investigator will submit a summary of the progress of the trial to the accredited METC once a year. Information will be provided on the date of inclusion of the first subject, numbers of subjects included and numbers of subjects that have completed the trial, serious adverse events/serious adverse reactions, other problems, and amendments.

12.5 End of study report

The investigator/sponsor will notify the accredited METC of the end of the study within a period of 8 weeks. The end of the study is defined as the last patient's last visit. The sponsor will notify the METC immediately of a temporary halt of the study, including the reason for such an action. In case the study is ended prematurely, the sponsor will notify the accredited METC within 15 days, including the reasons for the premature termination.

Within one year after the end of the study, the investigator/sponsor will submit a final study report with the results of the study, including any publications/abstracts of the study, to the accredited METC.

12.6 Public disclosure and publication policy

Not applicable

13. STRUCTURED RISK ANALYSIS

Risks of ingesting Potassium Iodide are minimal [10,11,12]. Potassium Iodide tablets of 65 mg are distributed among the population living within a certain distance from a nuclear power plant, with the advice to take a one-time dose of 130 mg in case of a nuclear disaster. The goal is to prevent the uptake of radioactive iodine and thereby prevent thyroid cancer. These tablets are distributed indiscriminately. This is justified because the benefit/risk ratio is high—in other words, the risk of possible mild side effects does not outweigh the risks of developing thyroid cancer.

In the pharmacotherapeutic compass, side effects are described for two indications for Potassium Iodide treatment. In addition to the aforementioned, Potassium Iodide is also given as preparation for thyroid surgery. Dosages used range from 150 to 750 mg per day for 10 to 14 days. The total dosage then amounts to 1,500 mg to 10,500 mg. Even at these dosages, relatively few and mild side effects are described.

Additionally, the website www.vergiftigingen.info does not advise additional measures for adults who have ingested a one-time dose of 10 × 65 mg = 650 mg Potassium Iodide tablets, since no relevant side effects are expected (see appendix 1).

The Potassium Iodide dosage used in our study—16.5 mg per day for 8 days, totaling 130 mg—is safe and the risk of side effects is very limited. The potential benefit of treatment with iodine is substantial, namely a less severe course of COVID-19 disease, a shorter illness period, a greater chance of survival, and possibly less irreversible lung damage.

13.1 Potential issues of concern

The chance of thyrotoxicosis occurring is very small. Should this nevertheless occur, the symptoms are temporary and transient.

a. **Level of knowledge about mechanism of action** The mechanism of thyrotoxicosis may cause hyperthyroidism due to autonomous dysregulation of the thyroid. It may also temporarily cause hypothyroidism due to blockage of iodine uptake by the thyroid.

b. **Previous exposure of human beings with the test product(s) and/or products with a similar biological mechanism**In previous studies, no relevant side effects were reported at the dosage and administration frequency used in our study [10,11,12].

c. **Can the primary or secondary mechanism be induced in animals and/or in ex-vivo human cell material?** Not known

d. **Selectivity of the mechanism to target tissue in animals and/or human beings** Not known

e. **Analysis of potential effect** Multiple in vitro studies and animal model studies have demonstrated virus inactivation by iodine.

f. **Pharmacokinetic considerations** Iodine is rapidly absorbed into the blood via the intestines. Iodine is actively taken up in the thyroid and is also relatively quickly excreted in various tissues such as mucous membranes.

g. **Study population** All patients aged 18 years and older who are admitted to the hospital and test positive for SARS-CoV-2 infection will be recruited for this study. Patients must be mentally competent and able to read the patient information sheet and sign the informed consent form.

h. **Interaction with other products** Not applicable

i. **Predictability of effect** It is expected that iodine will have a beneficial effect on the course of COVID-19 disease.

j. **Can effects be managed?** Should thyroid dysregulation occur in the very rare case, it is usually temporary and transient. If not, there are medications available to effectively treat this dysregulation.

13.2 Synthesis

The chance of medically relevant side effects is very limited at the dosage and administration frequency chosen in our study. Should this unexpectedly occur, the effects are transient or otherwise treatable with medication.

14. REFERENCES

1. Kelly F.C. (1961). Iodine in Medicine and Pharmacy since its Discovery 1811-1961. *Proceedings of the Royal Society of Medicine*, *54*(October) 831-836 https://doi.org/10.1177/003591576105401001

2. Derscheid, R. J., Van Geelen, A., Berkebile, A. R., Gallup, J. M., Hostetter, S. J., Banfi, B., McCray, P. B., & Ackermann, M. R. (2014). Increased concentration of iodide in airway secretions is associated with reduced respiratory syncytial virus disease severity. *American Journal of Respiratory Cell and Molecular Biology*, *50*(2), 389–397. https://doi.org/10.1165/rcmb.2012-0529OC

3. Fischer, A. J., Lennemann, N. J., Krishnamurthy, S., Pócza, P., Durairaj, L., Launspach, J. L., Rhein, B. A., Wohlford-Lenane, C., Lorentzen, D., Bánfi, B., & McCray, P. B. (2011). Enhancement of respiratory mucosal antiviral defenses by the oxidation of iodide. *American Journal of Respiratory Cell and Molecular Biology*, *45*(4), 874–881. https://doi.org/10.1165/rcmb.2010-0329OC

4. Blum, S. (1914). Iodine a specific germicide in respiratory affections: Preliminary Report. In *California state journal of medicine* Vol. 12, Issue 5, pp. 207–208. https://www.ncbi.nlm.nih.gov/pmc/articles/PMC1641102/

5. Menon, I. (1959). The 1957 pandemic of influenza in India. *Bulletin of the World Health Organization*. 20,199-224 https://www.ncbi.nlm.nih.gov/pmc/articles/PMC2537734/

6. World Health Organization. Coronavirus disease 2019. 2020. https://doi.org/10.1001/jama.2020.2633

7. Delange, F., De Benoist, B., Bürgi, H., Azizi, F., Hajipour, R., Benmiloud, M., Chen, Z. P., Dussault, J., Foo, L. C., Djokomoeljanto, R., Hartono, B., Hollowell, J. G., Irie, M., Jooste, P., Laurberg, P., Lozanov, B., Pretell, E., Rendl, J., Sinawat, S., … Zimmermann, M. (2002). Determining median urinary iodine concentration that indicates adequate iodine intake at population level. *Bulletin of the World Health Organization*, *80*(8), 633–636 https://doi.org/10.1590/S0042-96862002000800007

8. Olivieri, A., Di Cosmo, C., De Angelis, S., Da Cas, R., Stacchini, P., Pastorelli, A., & Vitti, P. (2017). The way forward in Italy for iodine. In *Minerva Medica*. Apr;108 (2):159-168 https://doi.org/10.23736/S0026-4806.17.04877-7

9. Olivieri, A., Tonacchera, M., & Vitti, P. (2012). Summary of the first report on the iodine nutritional status in Italy. *Iodine Global Netword Organization*, p 2–4. https://www.ign.org/cm_data/Summary_of_the_report_on_iodine_nutritional_status_in_Italy.pdf

10. Furnée, C.A. (1997). Prevention and Control of Iodine Deficiency: a review of a study on the effectiveness of oral iodized oil in Malai. European Journal of Clinical Nutrition S9-S10 https://pubmed.ncbi.nlm.nih.gov/9598786/

11. Kimball, O., & Marine, D. (1918). The prevention of simple goiter in man. Arch Int med, july;41-44 https://jamanetwork.com/journals/jamainternalmedicine/article-abstract/654242

12. Leverge, R., Bergmann, J. F., Simoneau, G., Tillet, Y., & Bonnemain, B. (2003). Bioavailability of oral vs intramuscular iodinated oil (Lipiodol UF) in healthy subjects. *Journal of Endocrinological Investigation.* 26(2 Suppl):20-26 https://pubmed.ncbi.nlm.nih.gov/12762636/?from_term=goiter+treatment+iodine+annual

13. Bruger, M., & Member, S. (1943). On the excretion of iodine in the saliva. *American Journal of Physiology-Legacy Content*.139 (2): 212-216 https://doi.org/10.1152/ajplegacy

14. Taylor, G. R., & Butler, M. (1982). A comparison of the virucidal properties of chlorine, chlorine dioxide, bromine chloride and iodine. *Journal of Hygiene.* (89), 321–328. https://doi.org/10.1017/S0022172400070856

15. McDonnell, G., & Russell, D. (1999). Antiseptics and disinfectants: activity, action, and resistance. *Clin. Microbiol. Rev. Jan.: 147–179* https://pubmed.ncbi.nlm.nih.gov/9880479/?from_term=Antiseptics+and+disinfectants%3A+activity%2C+action%2C+and+resistance&from_pos=1

16. Sriwilaijaroen, N., Wilairat, P., Hiramatsu, H., Takahashi, T., Suzuki, T., Ito, M., Ito, Y., Tashiro, M., & Suzuki, Y. (2009). Mechanisms of the action of povidone-iodine against human and avian influenza A viruses: Its effects on hemagglutination and sialidase activities. *Virol J.Aug 13; 6:124*. https://doi.org/10.1186/1743-422X-6-124

17. Eggers, M. (2019). Infectious Disease Management and Control with Povidone Iodine. *Infectious Diseases and Therapy*. (8):581-593 https://doi.org/10.1007/s40121-019-00260-x

18. Kenzhebekova, R. T., Abekova, A. O., Raziyeva, K. D., Abramova, Z. S., Islamov, R. A., Nersesyan, A. K., & Ilin, A. I. (2018). Investigation of the impact of iodine coordination compound on production of interleukin-4 and interferon-γ in vitro and primary evaluation of

local irritation in vivo. *Int. J. Biol. Chem. Vol 11, № 2, p.* 4–10. https://doi.org/10.26577/ijbch-2019-1-339

19. Sharma, S., Saimbi, C. S., Koirala, B., & Shukla, R. (2008). Effect of various mouthwashes on the levels of interleukin-2 and interferon-γ in chronic gingivitis. *Journal of Clinical Pediatric Dentistry*. 32 (2):111-114 https://doi.org/10.17796/jcpd.32.2.u01p135561161476

20. Snelgrove, R. J., Edwards, L., Rae, A. J., & Hussell, T.(2006) An absence of reactive oxygen species improves the resolution of lung influenza infection. Eur. J. Immunol. 36: 1364–1373 https://doi.org/10.1002/eji.200635977

21. Apostolov, K. (1980).The effects of iodine on the biological activities of myxoviruses. *Journal of Hygiëne*. 84, 381-388 https://doi.org/10.1017/S0022172400026905

22. Eggers, M., Koburger-Janssen, T., Eickmann, M., & Zorn, J. (2018). In Vitro Bactericidal and Virucidal Efficacy of Povidone-Iodine Gargle/Mouthwash Against Respiratory and Oral Tract Pathogens. *Infectious Diseases and Therapy*, *7*(2), 249–259. https://doi.org/10.1007/s40121-018-0200-7

23. Sattar, S. A., Springthorpe, V. S., Karim, Y., & Loro, P.(1989). Chemical disinfection of non-porous inanimate surfaces experimentally contaminated with four human pathogenic viruses. Epidemiology and Infection, 102(3), 493–505. https://doi.org/10.1017/S0950268800030211

24. Winkler, R. (2015). Iodine—A Potential Antioxidant and the Role of Iodine/Iodide in Health and Disease. Natural Science, 7, 548-557. https://doi.org/10.4236/ns.2015.712055

25. Shoemake, B. M., Vander Ley, B. L., Newcomer, B. W., & Heller, M. C. (2018). Efficacy of Oral Administration of Sodium Iodide to Prevent Bovine Respiratory Disease Complex. Journal of Veterinary Internal Medicine, 32(1), 516–524. https://doi.org/10.1111/jvim

26. Zou, L., SARS-CoV-2 viral load in upper respiratory specimens of infected patients. New England Journal of Medicine, (2020) 382(12), 1175–1177. https://doi.org/10.1056/NEJMc2000231

27. Sisk, J. et al. Coronavirus S protein-induced fusion is blocked prior to hemifusion by Abl kinase inhibitors. Journal of General Virology 2018; 99: 619–630 https://www.ncbi.nlm.nih.gov/pmc/articles/PMC6537626/pdf/jgv-99-619.pdf

28. Abdel-Mottaleb, M., In search for effective and safe drugs against SARS-CoV-2: Part II; The role of selected salts and organometallics of copper, zinc, selenium and iodine food supplements. (mei 5, 2020) https://chemrxiv.org/articles/In_Search_for_Effective_and_Safe_Drugs_Against_SARS-CoV-2_Part_II_the_Role_of_Selected_Salts_and_Organometallics_of_Copper_Zinc_Selenium_and_Iodine_Food_Supplements/12234743/1
