# Supplementary material for: Iodine increases pulmonary type I interferon responses and decreases covid-19 disease severity: Results from an open label randomized clinical trial
Source: PLoS One. 2026 Feb 2;21(2):e0341126. doi: 10.1371/journal.pone.0341126 (PMC12863515; doi:10.1371/journal.pone.0341126)
Supplement: S2 File — (DOCX) [file pone.0341126.s005.docx]

**Figure 1: CONSORT 2025 Flow Diagram**

Flow diagram of the progress through the phases of a randomised trial of two groups (that is, enrolment, intervention allocation, follow-up, and data analysis)

Randomised (n=150 )

Analysis

Analysed for primary outcome (n= 74 )

Excluded from analysis (give reasons) (n= 0)

Discontinued intervention (give reasons) (n=0 )

Lost to follow-up for primary outcome (give reasons) (n= 0):

Discontinued intervention (give reasons) (n=9 ) Patients moved to other facility, missed dosing at ward

Lost to follow-up for primary outcome (give reasons) (n= 0):

Excluded (n= 54)

Not meeting inclusion criteria (n= 0)

Declined to participate (n= 54)

Other reasons (n= )

Allocation

Follow-Up

Allocated to standard care (n= 74)

Received allocated intervention (n= 74)

Did not receive allocated intervention (give reasons) (n= 0)

Allocated to standard care+iodine (n=76 )

Received allocated intervention (n=76 )

Did not receive allocated intervention (give reasons) (n= 0)

Enrolment

Assessed for eligibility (n= 204)

Analysed for primary outcome (n= 67)

Excluded from analysis (give reasons) (n= 0)
