# Supplementary material for: Iodine increases pulmonary type I interferon responses and decreases covid-19 disease severity: Results from an open label randomized clinical trial
Source: PLoS One. 2026 Feb 2;21(2):e0341126. doi: 10.1371/journal.pone.0341126 (PMC12863515; doi:10.1371/journal.pone.0341126)
Supplement: S3 File — (PDF) [file pone.0341126.s006.pdf]

RESEARCH PROTOCOL

Effect van Jodium behandeling bij patienten met COVID-19 infectie

(juni 2020)

PROTOCOL TITLE 'Effect Iodine treatment in patients with COVID-19 infection'

**Protocol ID**	**N20.0490**
**Short title**	**Jodium behandeling Coronavirus**
**EudraCT number**	**2020-001852-16**
**Version**	**3**
**Date**	**20200603**
**Coordinating investigator/project leader**	***R.A.M. Traksel Maxima MC, r.traksel@mmc.nl***
**Principal investigator(s) (in Dutch: hoofdonderzoeker/ uitvoerder)**	***R.A.M. Traksel Maxima MC, r.traksel@mmc.nl*** ***R.H. Verheesen Maxima MC, rh.verheesen@mmc.nl*** ***J.C.A. Broen Maxima MC, j.broen@mmc.nl***
**Sponsor (in Dutch: verrichter/opdrachtgever)**	***Maxima MC***
**Subsidising party**	
**Independent expert (s)**	***M.Y. Bongers, Maxima MC, m.bongers@mmc.nl***
**Laboratory sites <*if applicable*>**	***Niet van toepassing***
**Pharmacy**	**P.A.G. de Klaver, Maxima MC, *p.deKlaver@mmc.nl***

PROTOCOL SIGNATURE SHEET

Name	Signature	Date
R.A.M. Traksel		13-7-2020
R.H. Verheesen		
J.C.A. Broen		
P.A.G. de Klaver		11-07-2020

TABLE OF CONTENTS

1. INTRODUCTION AND RATIONALE 8
2. OBJECTIVES 9
3. STUDY DESIGN 9
4. STUDY POPULATION 9
   4.1 Population (base) 11
   4.2 Inclusion criteria 9
   4.3 Exclusion criteria 9
   4.4 Sample size calculation 9
5. TREATMENT OF SUBJECTS 10
   5.1 Investigational product/treatment 10
   5.2 Use of co-intervention (if applicable) 10
   5.3 Escape medication (if applicable) 10
6. INVESTIGATIONAL PRODUCT 10
   6.1 Name and description of investigational product(s) 10
   6.2 Summary of findings from non-clinical studies 10
   6.3 Summary of findings from clinical studies 10
   6.4 Summary of known and potential risks and benefits 10
   6.5 Description and justification of route of administration and dosage 10
   6.6 Dosages, dosage modifications and method of administration 11
   6.7 Preparation and labelling of Investigational Medicinal Product 11
   6.8 Drug accountability 11
7. NON-INVESTIGATIONAL PRODUCT 11
   7.1 Name and description of non-investigational product(s) 12
   7.2 Summary of findings from non-clinical studies 12
   7.3 Summary of findings from clinical studies 12
   7.4 Summary of known and potential risks and benefits 12
   7.5 Description and justification of route of administration and dosage 12
   7.6 Dosages, dosage modifications and method of administration 12
   7.7 Preparation and labelling of Non Investigational Medicinal Product 12
   7.8 Drug accountability 12
8. METHODS 12
   8.1 Study parameters/endpoints 12
      8.1.1 Main study parameter/endpoint 12
      8.1.2 Secondary study parameters/endpoints (if applicable) 12
      8.1.3 Other study parameters (if applicable) 15
   8.2 Randomisation, blinding and treatment allocation 12
   8.3 Study procedures 12
   8.4 Withdrawal of individual subjects 12
      8.4.1 Specific criteria for withdrawal (if applicable) 13
   8.5 Replacement of individual subjects after withdrawal 13
   8.6 Follow-up of subjects withdrawn from treatment 13

8.7 Premature termination of the study 13
9. SAFETY REPORTING 13
9.1 Temporary halt for reasons of subject safety 16
9.2 AEs, SAEs and SUSARs 13
9.2.1 Adverse events (AEs) 13
9.2.2 Serious adverse events (SAEs) 13
9.2.3 Suspected unexpected serious adverse reactions (SUSARs) 14
9.3 Annual safety report 14
9.4 Follow-up of adverse events 14
9.5 [Data Safety Monitoring Board (DSMB) / Safety Committee] 14
10. STATISTICAL ANALYSIS 15
10.1 Primary study parameter(s) 15
10.2 Secondary study parameter(s) 15
10.3 Other study parameters 15
10.4 Interim analysis (if applicable) 15
11. ETHICAL CONSIDERATIONS 16
11.1 Regulation statement 16
11.2 Recruitment and consent 16
11.3 Objection by minors or incapacitated subjects (if applicable) 16
11.4 Benefits and risks assessment, group relatedness 16
11.5 Compensation for injury 16
11.6 Incentives (if applicable) 16
12. ADMINISTRATIVE ASPECTS, MONITORING AND PUBLICATION 17
12.1 Handling and storage of data and documents 17
12.2 Monitoring and Quality Assurance 17
12.3 Amendments 17
12.4 Annual progress report 17
12.5 End of study report 17
12.6 Public disclosure and publication policy 17
13. STRUCTURED RISK ANALYSIS 18
13.1 Potential issues of concern 18
13.2 Synthesis 20
14. REFERENCES 21

LIST OF ABBREVIATIONS AND RELEVANT DEFINITIONS

ABR	General Assessment and Registration form (ABR form), the application form that is required for submission to the accredited Ethics Committee; in Dutch: Algemeen Beoordelings- en Registratieformulier (ABR-formulier)
AE	Adverse Event
AR	Adverse Reaction
CA	Competent Authority
CCMO	Central Committee on Research Involving Human Subjects; in Dutch: Centrale Commissie Mensgebonden Onderzoek
CV	Curriculum Vitae
DSMB	Data Safety Monitoring Board
EU	European Union
EudraCT	European drug regulatory affairs Clinical Trials
GCP	Good Clinical Practice
GDPR	General Data Protection Regulation; in Dutch: Algemene Verordening Gegevensbescherming (AVG)
IB	Investigator's Brochure
IC	Informed Consent
IMP	Investigational Medicinal Product
IMPD	Investigational Medicinal Product Dossier
METC	Medical research ethics committee (MREC); in Dutch: medisch-ethische toetsingscommissie (METC)
(S)AE	(Serious) Adverse Event
SPC	Summary of Product Characteristics; in Dutch: officiële productinformatie IB1-tekst
Sponsor	The sponsor is the party that commissions the organisation or performance of the research, for example a pharmaceutical company, academic hospital, scientific organisation or investigator. A party that provides funding for a study but does not commission it is not regarded as the sponsor, but referred to as a subsidising party.
SUSAR	Suspected Unexpected Serious Adverse Reaction
UAVG	Dutch Act on Implementation of the General Data Protection Regulation; in Dutch: Uitvoeringswet AVG
WMO	Medical Research Involving Human Subjects Act; in Dutch: Wet Medisch-wetenschappelijk Onderzoek met Mensen

SUMMARY

Rationale:
Jodium heeft een germicide werking en levert een bijdrage aan het afweermechanisme tegen pathogenen. Daarnaast heeft Jodium anti-inflammatoire en immuun modulerende eigenschappen die een gunstig effect kunnen hebben bij de behandeling van COVID-19 infecties. Dit leidt tot onze hypothese dat jodium effectief is bij de behandeling van COVID-19 ziekte.

Objective:
Aan tonen dat jodium behandeling een gunstig effect heeft op het beloop van COVID-19 ziekte. Gunstig effect is gedefinieerd als minder kans op een verslechtering zoals overplaatsing van gewone verpleegafdeling naar IC afdeling, overlijden op gewone verpleegafdeling of overlijden op IC afdeling.

Study design:
Open label gerandomiseerd onderzoek met controle groep

Study population:
Alle patienten van 18 jaar en ouder, die worden opgenomen in het ziekenhuis en positief testen voor SARS-CoV-2 infectie worden geworven voor het onderzoek. Patienten moeten wilsbekwaam zijn en in staat om de patienten informatiebrief te lezen en het informed consent formulier te ondertekenen.

Intervention:
Patiënten in de interventie groep krijgen 8 aan een gesloten dagen, 1 x daags ¼ Kaliumjodide 65 mg tablet. De controle groep krijgt geen supplement en geen placebo.

Main study parameters/endpoints:
Primaire eindpunt: klinisch relevante verslechtering.
Verslechtering is gedefinieerd als:

1. Van baseline op gewone verpleegafdeling naar IC afdeling
2. Van baseline op gewone verpleegafdeling en overlijden
3. Van baseline op IC afdeling en overlijden

Secundair eindpunt: totaal aantal dagen ziekenhuis opname en totaal aantal overlijden

Nature and extent of the burden and risks associated with participation, benefit and group relatedness:
Wanneer patiënt wordt gerandomiseerd en in de behandelgroep komt dan is er een gunstig effect te verwachten op het beloop van de COVID-19 infectie. Patiënt dient dan 8 dagen achter elkaar, 1 x daags ¼ Kaliumjodide 65 mg tablet te gebruiken. Het risico op bij het hanteren van de exclusie criteria is zeer beperkt en in principe van tijdelijke aard.

1. INTRODUCTION AND RATIONALE

Jodium heeft een germicide werking en levert een bijdrage aan het afweermechanisme tegen pathogenen **[1]**. Het SARS-CoV-2 virus heeft naast een beschermende eiwitmantel, de capside, nog een buitenste lipide membraan de envelop, waarin tevens eiwitten zijn verankerd. Hemagglutinine (HA) is een oppervlakte-eiwit dat zorgt voor herkenning van de gastheercellen en voor "injectie" van het virale genoom door het samensmelten van de endosomale membranen te veroorzaken. Neuraminidase (NA) is een ander oppervlakte-eiwit dat er voor zorgt dat virusdeeltjes, na vermenigvuldiging in cellen, van deze cellen kunnen loskoppelen en zich verder kunnen verspreiden. Dit doet het door siaalzuurresidu's af te knippen. Jodium vermindert de virulentie van virussen door interactie met verschillende oppervlakte-eiwitten. Dit leidt tot degeneratie van de capside en daarmee tot virus inactivatie **[14]**. Daarnaast werkt Jodium inhiberend op de HA en NA activiteit waardoor het binnendringen van de gastheercel respectievelijk het verspreiden van virusdeeltjes buiten de cel bemoeilijkt wordt **[16,17,21]**. Tevens kan jodium mogelijk een destabilisatie van de envelop veroorzaken door een reactie met de onverzadigde koolstofverbindingen **[15]**. Jodium heeft antivirale eigenschappen tegen Coronavirussen **[22,23]**. Recent is er een chemische interactie beschreven van jodium met het Coronavirus spike (S) eiwit, dat fusie van het virus met de gastheercel faciliteert, en met de ACE2 receptoren. Deze bevindingen suggereren sterk een gunstig effect van jodium behandeling bij Coronavirus infecties **[27,28]**. COVID-19 ziekte kan dodelijk zijn als gevolg van een overreactie van het immuunsysteem van het lichaam, een cytokine-storm genaamd. Er worden dan meer pro-inflammatoire cytokines geproduceerd zoals IL-2, IL-8 en IFN-gamma en TNF alfa. Behandeling met Jodium leidt bij niet toxische doseringen tot afname van deze cytokines **[2,18,19]**. Daarnaast wordt er een overmaat aan Reactive Oxygen Species ROS (radicalen) geproduceerd die toxisch zijn voor pathogenen maar die tegelijkertijd ook schadelijk kunnen zijn voor het lichaam zelf **[20]**. Het wegvangen van deze overmaat aan radicalen door antioxidanten kan een gunstig effect hebben op het beloop van aandoeningen die gepaard gaan met hoge oxidatieve stress **[20]**. Jodium is een sterke antioxidant en vangt een overmaat aan ROS weg **[3,24]**.

Jodium wordt via de slijmvliezen in de mond en trachea uit gescheiden deels via Natrium-Jodium symporters. Verder is er een relatie tussen de jodium status en concentratie van Jodium in speeksel **[13,25]**. Daarnaast heeft Jodium anti-inflammatoire en immuun modulerende eigenschappen die een gunstig effect kunnen hebben bij de behandeling van COVID-19 infecties **[2,3]**. In meerdere case report wordt een gunstig effect van Jodium behandeling bij virale infecties beschreven **[4,5]**.

Bij epidemiologisch onderzoek naar aantal overleden COVID-19 patiënten wordt een opvallen verschil gevonden tussen Japan en Italië. Op 1 april 2020 telt Japan 57 doden met 126 miljoen inwoners (0,5/mln.) en Italië 12.428 doden met 60 miljoen inwoners (207/mln.) **[6]**. Verder staan Japanners bekend om hun hoge jodium inname via de voeding in tegenstelling tot Italianen die jodium deficiënt zijn. De jodium status, gemeten in Urine Jodium Concentratie(UIC) volgens de WHO criteria, bedraagt in Japan 282 mcg/L en in Italië 84 mcg/L **[7,8,9]**. Deze twee bevindingen suggereren een beschermend effect van jodium tegen COVID-19 infecties. Dit leidt tot onze hypothese dat jodium effectief is bij de behandeling van COVID-19 infecties. Om de hypothese te toetsen willen wij onderzoek doen naar de behandeling van Jodium bij COVID-19 geïnfecteerde patiënten.

2. OBJECTIVES
Primaire eindpunt: klinisch relevante verslechtering.
Verslechtering is gedefinieerd als:

a. Van baseline op gewone verpleegafdeling naar IC afdeling
b. Van baseline op gewone verpleegafdeling en overlijden
c. Van baseline op IC afdeling en overlijden

Secundair eindpunt:

a. totaal aantal dagen ziekenhuis opname
b. totaal aantal overlijden

3. STUDY DESIGN
Open label gerandomiseerd onderzoek met controle groep.

4. STUDY POPULATION
4.1 Populatie
Opgenomen patienten met COVID-19 ziekte in Maxima MC

4.2 Inclusion criteria
Alle patienten van 18 jaar en ouder, die positief testen op SARS-CoV-2 infectie kunnen mee doen aan dit onderzoek.

4.3 Exclusion criteria
Schildklieraandoening of behandeling zoals struma, thyreoidectomie, radioactief jodium, medicijnen in verband met schildklierdysfunctie of Amiodaron
De onderzoeker moet toegang hebben tot de medische voorgeschiedenis en medicatie gebruik van proefpersoon. Waar nodig zal contact worden opgenomen met de huisarts of andere ziekenhuizen om de benodigde informatie te verkrijgen. Indien geen of onvoldoende gegevens verkregen kunnen worden dan kan de proefpersoon niet mee doen aan het onderzoek.

4.4 Sample size calculation
Indien een minder optimistisch scenario wordt geschetst waarbij wordt uitgegaan van 25% verslechtering (overplaatsing naar IC of overlijden op afdeling of op IC) in de controle-arm en 10% in de interventie groep dan wordt de powerberekening als volgt:
25% vs 10% > 100 pp per groep > 200 pp totaal > 10% uitval > 224 pp nodig

Er is een interim analyse gepland met stopregel volgens O'Brien Fleming, waarvoor geen aanpassing van de sample size nodig is. Na 50 patienten in iedere arm

5. TREATMENT OF SUBJECTS

Gedurende 8 aan een gesloten dagen, 1 x daags ¼ Kaliumjodide 65 mg tablet. Totale dosering Jodium tijdens de kuur behandeling bedraagt 130 mg. Na 8 dagen wordt er een forse afname van SARS-CoV-2 virus titers in neus- en keeluitstrijk **[26].**

5.1 Investigational product/treatment

Kaliumjodide 65 mg tablet

5.2 Use of co-intervention (if applicable)

Patienten krijgen de zelfde standard behandeling als andere patienten met een COVID-19 infectie. Er zijn geen restricties voor het gebruik van ander medicatie

5.3 Escape medication (if applicable)

Niet van toepassing

6 INVESTIGATIONAL PRODUCT

6.1 Name and description of investigational product

Kaliumjodide 65 mg tablet

6.2 Summary of findings from non-clinical studies

Geen studies bekend

6.3 Summary of findings from clinical studies

Geen studies bekend

6.4 Summary of known and potential risks and benefits

Kans op bijwerking wordt herleid van de vermeldingen in het farmacotherapeutisch kompas bij de Kaliumjodide tabletten van 65 mg.

De daar vermelde bijwerking zijn relatief mild en in principe van voorbijgaande aard. In zeldzame gevallen bij pre-existente schildklier aandoeningen zou zich een thyreotoxicose zich voor kunnen doen. Dan kan tijdelijk medicamenteuze behandeling zijn ter correctie van een schildklier dysfunctie nodig zijn. Dit wordt echter niet verwacht aangezien een schildklier aandoening of medicatie ivm schildklierdysfunctie een exclusie criterium is. Uit eerdere studies blijkt dat het toedienen van relatief hoge dosering Jodium veilig is. **[10,11,12]**

6.5 Description and justification of route of administration and dosage

Orale toediening gedurende 8 dagen 1 x daags ¼ Kaliumjodide 65 mg tablet is weinig belastend voor patiënt en eenvoudig toe te dienen door verpleegkundige. Verder zijn de tablet erg goedkoop € 0,01 per tablet volgens opgave farmacotherapeutisch kompas.

Uit meerdere in vitro studies en diermodel onderzoek komen verschillende benodigde concentraties om virus inactivatie te bereiken. Hierbij is vooral gekeken naar virussen met en envelop omdat het SARS-CoV-2 virus ook een envelop heeft.

Benodigde concentraties voor envelop virus inactivatie variëren tussen de 10 µM en 80 µM Jodium. [2, 3, 7, 21, 22]

Om tot een inschatting te komen van de benodigde dosering zijn de volgende aannames gedaan:

- De jodium concentratie in het alveolaire vocht is 20 X zo groot als in het serum **[2]**. Door actief transport van jodium naar het lumen via Natrium Jodium Symporters in de mucosa kan dit concentratie verschil gerealiseerd worden.

- Voor een gemiddeld gewicht van 80 kg bedraagt de hoeveelheid lichaamswater ongeveer 50 liter.
- Van de toegediende dagelijkse ¼ Kaliumjodide 65 mg tablet wordt 100% (16,5 mg) opgenomen
- De halfwaardetijd van jodium gemeten in **alveolaire longvocht** na éénmalige Kaliumjodide toediening in diermodel bedraagt ongeveer 24 uur **[2].** Na eenmalige inname van Kaliumjodide zal de jodiumspiegel **in serum** binnen 24 tot 48 uur weer op baseline niveau terug zijn **[24].**
- Mol massa Kalium is 40 g en Mol massa Jodium is 127 g gezamenlijk 167 g. Dus in 16,5 mg Kaliumjodide zit (127/167) x 16,5 mg = 12,5 mg Jodium. Dit komt over een met (0,0125/127) = 98 µmol jodium.
- Bij een volume van 50 liter lichaamswater bedraagt de concentratie van Jodium 98/50 = 2 µM
- Van de totale hoeveelheid jodium in het lichaam is de helft, 6 mg tot 14 mg, opgeslagen in de schildklier**[23].** Van de andere helft wordt verondersteld dat dit perifeer beschikbaar is. Dus in het lichaam zelf is 6 tot 14 mg Jodium beschikbaar in 50 liter lichaamswater. Dit komt overeen met 6/50 = 0,12 mg (0,94 µmol) respectievelijk 14/50 = 0,28 mg (2,2 µmol) per liter.
- De concentratie berekening in serum is dan de optelsom van aanwezig jodium in serum + de extra jodium toevoeging dus 0,94 + 2 = 2,94 µM respectievelijk 2,2 + 2 = 4,2 µM.
- De Jodium concentratie in het alveolaire vocht is 20 x zo hoog als in het serum. **Dus de berekende uitersten jodium concentratie in serum van 2,94 µM en 4,2 µM komt overeen met 2,94 x 20 = 58,8 µM respectievelijk 4,2 x 20 = 84 µM in alveolaire longvloeistof**.
- In de literatuur aangegeven doseringen die noodzakelijk zijn voor inactiviteit van envelop virussen, ligt **tussen de 10 µM en 80 µM.**
- Zoals uit onze berekening blijkt ligt onze dagelijkse dosering van ¼ Kaliumjodide 65 mg tablet binnen de range van de benodigde Jodium concentraties, voor inactivatie van envelop virussen, op alveolair longvloeistof niveau.
- De jodium toediening duurt 8 dagen omdat je pas na een week duidelijke afname ziet van SARS-CoV-2 virus titers **[25]**. Daarnaast geven we jodium niet alleen voor de antivirale eigenschappen maar ook in verband met de antioxidanten werking van jodium dat nodig is om de ontstane vrije radicalen bij een mogelijk aanwezige cytokine storm te adresseren. Derhalve hebben we gekozen voor een behandel duur van 8 dagen. De totale dosering van de kuur komt dan op 130 mg Kaliumjodide.

6.6 Dosages, dosage modifications and method of administration

Gedurende 8 dagen achter elkaar, 1 x daags ¼ Kaliumjodide 65 mg tablet. Verstrekkingen worden door de verpleegkundige geregistreerd in het elektronisch voorschrijf systeem van het EPD van het ziekenhuis.

6.7 Preparation and labelling of Investigational Medicinal Product

Het betreft een beschikbaar geneesmiddel dat via apotheek van het ziekenhuis verstrekt zal worden

6.8 Drug accountability

Kaliumjodide zal alleen tijdens het verblijf in het ziekenhuis worden verstrekt. Het voorschrift wordt in het elektronisch voorschrijfsysteem van het EPD van het ziekenhuis vermeld.

7. NON-INVESTIGATIONAL PRODUCT

Niet van toepassing

7.1 Name and description of non-investigational product(s)

7.2 Summary of findings from non-clinical studies

7.3 Summary of findings from clinical studies

7.4 Summary of known and potential risks and benefits

7.5 Description and justification of route of administration and dosage

7.6 Dosages, dosage modifications and method of administration

7.7 Preparation and labelling of Non Investigational Medicinal Product

7.8 Drug accountability

8. METHODS

8.1 Study parameters/endpoints

8.1.1 Main study parameter/endpoint

Primaire eindpunt: klinisch relevante verslechtering.
Verslechtering is gedefinieerd als:

a. Van baseline op gewone verpleegafdeling naar IC afdeling
b. Van baseline op gewone verpleegafdeling en overlijden
c. Van baseline op IC afdeling en overlijden

8.1.2 Secondary study parameters/endpoints

Secundair eindpunt:

d. Totaal aantal overlijden
e. Totaal aantal dagen opname in het ziekenhuis

Als de proefpersoon een deel van een kalenderdag op de intensive care afdeling opgenomen is geweest dan telt deze dag als 1 hele dag opname op de intensive care afdeling. Als de proefpersoon op een kalenderdag deels op de IC en deels op een verpleegafdeling opgenomen is geweest dan telt deze dag als 1 hele dag IC opname. Als de patient een deel van een kalenderdag op een verpleegafdeling opgenomen is geweest en op die dag is overleden of ontslagen dan telt deze dag als 1 hele dag verpleegafdeling opname.

8.2 Randomisation, blinding and treatment allocation

Randomisation via Research manager

8.3 Study procedures

Patient informatie formulier doornemen en informed consent ondertekenen.
Inclusie periode loopt van 1 juli 2020 tot 1 juli 2022.

8.4 Withdrawal of individual subjects

Proefpersonen kunnen het onderzoek op elk moment en om welke reden dan ook stoppen, zonder dat daar consequenties aan verbonden zijn. De onderzoeker kan om dringende medische redenen besluiten een proefpersoon uit het onderzoek terug te trekken.

8.4.1 Specific criteria for withdrawal (if applicable)

8.5 Replacement of individual subjects after withdrawal
Er is rekening gehouden met enige uitval bij de power berekening. Er vindt geen vervanging plaats.

8.6 Follow-up of subjects withdrawn from treatment
Follow up duurt zolang als patient is opgenomen in het ziekenhuis.

8.7 Premature termination of the study
Onverwachte ernstige bijwerkingen kan een reden zijn om het onderzoek te stoppen. Toediening van Kaliumjodide tablet zal dan direct gestaakt worden.

9. SAFETY REPORTING

9.1 Temporary halt for reasons of subject safety
In accordance to section 10, subsection 4, of the WMO, the sponsor will suspend the study if there is sufficient ground that continuation of the study will jeopardise subject health or safety. The sponsor will notify the accredited METC without undue delay of a temporary halt including the reason for such an action. The study will be suspended pending a further positive decision by the accredited METC. The investigator will take care that all subjects are kept informed.

9.2 AEs, SAEs and SUSARs

9.2.1 Adverse events (AEs)
Adverse events are defined as any undesirable experience occurring to a subject during the study, whether or not considered related to [the investigational product / trial procedure/ the experimental intervention]. All adverse events reported spontaneously by the subject or observed by the investigator or his staff will be recorded. Deze informatie wordt opgeslagen in het EPD van de proefpersonen

9.2.2 Serious adverse events (SAEs)
A serious adverse event is any untoward medical occurrence or effect that

- results in death;
- is life threatening (at the time of the event
- requires hospitalisation or prolongation of existing inpatients' hospitalisation;
- results in persistent or significant disability or incapacity;
- is a congenital anomaly or birth defect; or
- any other important medical event that did not result in any of the outcomes listed above due to medical or surgical intervention but could have been based upon appropriate judgement by the investigator.

An elective hospital admission will not be considered as a serious adverse event.

The investigator will report all SAEs to the sponsor without undue delay after obtaining knowledge of the events, except for the following SAEs: niet van toepassing

The sponsor will report the SAEs through the web portal *ToetsingOnline* to the accredited METC that approved the protocol, within 7 days of first knowledge for

SAEs that result in death or are life threatening followed by a period of maximum of 8 days to complete the initial preliminary report. All other SAEs will be reported within a period of maximum 15 days after the sponsor has first knowledge of the serious adverse events.

9.2.3 Suspected unexpected serious adverse reactions (SUSARs)

9.3 Annual safety report

In addition to the expedited reporting of SUSARs, the sponsor will submit, once a year throughout the clinical trial, a safety report to the accredited METC, competent authority, and competent authorities of the concerned Member States.

This safety report consists of:

- a list of all suspected (unexpected or expected) serious adverse reactions, along with an aggregated summary table of all reported serious adverse reactions, ordered by organ system, per study;
- a report concerning the safety of the subjects, consisting of a complete safety analysis and an evaluation of the balance between the efficacy and the harmfulness of the medicine under investigation.

9.4 Follow-up of adverse events

All AEs will be followed until they have abated, or until a stable situation has been reached. Depending on the event, follow up may require additional tests or medical procedures as indicated, and/or referral to the general physician or a medical specialist. SAEs need to be reported till end of study within the Netherlands, as defined in the protocol

9.5 [Data Safety Monitoring Board (DSMB) / Safety Committee]

10. STATISTICAL ANALYSIS

Primaire analyse van het eindpunt "klinisch relevante verslechtering" is een Kaplan-Meier survival analyse met als looptijd het aantal dagen sinds studiestart. Studie start is kalenderdag waarop eerste gift Kaliumjodide tablet is verstrekt. Het verschil in overplaatsing van gewone afdeling naar IC afdeling, overlijden op gewone verpleegafdeling of overlijden op IC afdeling, wordt getoetst met een log-rank toets. Daarnaast worden de hazards ratio's met 95%CI berekenen met behulp van Cox-regressie analyse.

Secundaire analyse wordt verricht voor alléén overlijden en alléén het totaal aantal dagen opname in het ziekenhuis. Hiervoor zal een Kaplan-Meier met Log-rank toets en Cox-regressie analyse verricht worden.

Primaire analyses

Primaire eindpunt: klinisch relevante verslechtering. Verslechtering is gedefinieerd als:

a. Van baseline op gewone verpleegafdeling naar IC afdeling
b. Van baseline op gewone verpleegafdeling en overlijden
c. Van baseline op IC afdeling en overlijden

Secundaire analyses

Secundair eindpunt:

d. Totaal aantal overlijden
e. Totaal aantal dagen opname in het ziekenhuis

10.1 Primary study parameter(s)

Klinisch relevante verslechtering uit gedrukt in aantal patienten die van een gewone afdeling worden overgeplaatst naar de IC afdeling en het aantal patienten dat overlijd op de gewone afdeling of op de IC afdeling.

10.2 Secondary study parameter(s)

Totaal aantal overlijden en het totaal aantal dagen opname in het ziekenhuis

10.3 Other study parameters

BSE, CRP, leukocyten, ferritine, zuurstof saturatie bij opname en temperatuur verloop. Deze metingen en onderzoeken worden gedaan in het kader van reguliere zorg.

10.4 Interim analysis

Halverwege de studie, bij 50 inclusies per arm zal een interim analyse worden uitgevoerd. Er zijn 2 redenen waarom de studie vroegtijdig gestopt kan worden.

1. Vanwege futiliteit. De studie zal worden gestopt indien bij herberekening van de sample size meer dan 100 deelnemers per arm nodig zijn of het verschil tussen controle en interventie arm niet klinische relevant wordt beschouwd (i.e. minder dan 5% verschil).

2. Vanwege effectiviteit (stopregel van O'Brien Fleming). De studie wordt gestopt bij een p-waarde<0.0054 voor de null-hypothese (i.e. geen verschil tussen interventie en referentie).

11. ETHICAL CONSIDERATIONS

11.1 Regulation statement

The study will be conducted according to the principles of the Declaration of Helsinki (version, date, see for the most recent version: www.wma.net) and in accordance with the Medical Research Involving Human Subjects Act (WMO) and other guidelines, regulations and Acts

11.2 Recruitment and consent

Patienten worden geworven door verpleegkundige, physician assistant io of behandelend arts. Toestemming wordt gevraagd door de onderzoeker.

11.3 Objection by minors or incapacitated subjects (if applicable)

Patienten die mee willen doen moeten 18 jaar of ouder en wilsbekwaam zijn

11.4 Benefits and risks assessment, group relatedness

Kans op bijwerking wordt herleid van de vermeldingen in het farmacotherapeutisch kompas bij de Kaliumjodide tabletten van 65 mg. De daar vermelde bijwerking zijn relatief mild en in principe van voorbijgaande aard. In zeldzame gevallen bij pre-existente schildklier aandoeningen zou zich een thyreotoxicose zich voor kunnen doen. Dan kan tijdelijk medicamenteuze behandeling zijn ter correctie van een schildklier dysfunctie nodig zijn. Dit wordt echter niet verwacht aangezien een schildklier aandoening of medicatie ivm schildklierdysfunctie een exclusie criterium is. Het risico op bijwerkingen bij de dosering Kaliumjodide zoals door ons gebruikt in dit onderzoek is zeer beperkt. Door schildklieraandoeningen en medicatie gebruik ivm schildklierdysfunctie als exclusie criterium te hanteren is de kans op een thyreotoxicose vrijwel uitgesloten. De overige bijwerkingen zijn relatief gering en doorgaans van voorbijgaande aard. Er zijn bij deze kortdurende Kaliumjodide behandeling geen gezondheidsrisico's voor de lange termijn te verwachten. De potentiele voordelen voor de patiënt kan aanzienlijk zijn, zoals een betere overlevingskans, een sneller herstel en een kortere duur van de ziekenhuisopname. Verder is er mogelijk minder irreversibele longschade ontstaan zodat de kwaliteit van leven na de ziekte periode beter zal zijn.

11.5 Compensation for injury

Proef personen verzekering is aangevraagd

11.6 Incentives

Niet van toepassing

12. ADMINISTRATIVE ASPECTS, MONITORING AND PUBLICATION

12.1 Handling and storage of data and documents

Research manager wordt gebruikt in een beveiligde ziekenhuis omgeving om gegevens op te slaan. Broncode wordt in een beveiligd Excel bestand opgeslagen.

12.2 Monitoring and Quality Assurance

Gezien de korte duur van de studie en zal praktisch gekeken worden naar hoe dit op afstand is te organiseren. Er is contact geweest met CTCM en een eerste visite heeft telefonisch plaats gevonden op 16 april.

12.3 Amendments

Amendments are changes made to the research after a favourable opinion by the accredited METC has been given. All amendments will be notified to the METC that gave a favourable opinion.
Non-substantial amendments will not be notified to the accredited METC and the competent authority, but will be recorded and filed by the sponsor.

12.4 Annual progress report

The sponsor/investigator will submit a summary of the progress of the trial to the accredited METC once a year. Information will be provided on the date of inclusion of the first subject, numbers of subjects included and numbers of subjects that have completed the trial, serious adverse events/ serious adverse reactions, other problems, and amendments.

12.5 Temporary halt and (prematurely) end of study report

The investigator/sponsor will notify the accredited METC of the end of the study within a period of 8 weeks. The end of the study is defined as the last patient's last visit.

The sponsor will notify the METC immediately of a temporary halt of the study, including the reason of such an action.

In case the study is ended prematurely, the sponsor will notify the accredited METC within 15 days, including the reasons for the premature termination.

Within one year after the end of the study, the investigator/sponsor will submit a final study report with the results of the study, including any publications/abstracts of the study, to the accredited METC.

12.6 Public disclosure and publication policy

Niet van toepassing

13. STRUCTURED RISK ANALYSIS

Risico's van inname van Kaliumjodide zijn minimaal **[10,11,12]**. Kaliumjodide tabletten van 65 mg wordt onder de bevolking, die binnen een bepaalde afstand van een kerncentrale woont, verspreid met het advies om bij een kernramp éénmalig 130 mg in te nemen. Het doel is om de opname van radioactief jodium, en daarmee schildklierkanker te voorkomen. Deze tabletten worden zonder aanziens des persoons verspreid. Dit is verantwoord omdat het benefit/risk ratio groot is met andere woorden het risico op mogelijke milde bijwerking wegen niet op tegen de risico's van het ontwikkelen van schildklierkanker.
In het farmacotherapeutisch kompas worden bijwerkingen beschreven bij twee indicaties voor Kaliumjodide behandeling. Naast de hiervoor genoemde wordt Kaliumjodide ook gegeven als voorbereiding op een schilklieroperatie. Daarbij worden doseringen gehanteerd van 150 tot 750 mg per dag gedurende 10 tot 14 dagen. De totale dosering komt dan op 1.500 mg tot 10.500 mg. Ook bij deze doseringen worden relatief weinig en milde bijwerkingen beschreven.
Daarnaast wordt op de website www.vergiftigingen.info geen aanvullende maatregelen geadviseerd bij volwassenen die éénmalig 10 x 65 mg = 650 mg Kaliumjodide tabletten hebben ingenomen aangezien geen relevante bijwerkingen verwacht worden (zie bijlage 1).
De Kaliumjodide dosering die wij in onze studie gebruiken van 16,5 mg per dag gedurende 8 dagen, in totaal 130 mg, is veilig en het risico op bijwerking zeer beperkt. Het potentieel voordeel van een behandeling met jodium is groot namelijk een minder ernstig beloop van de COVID-19 ziekte, een kortere ziekte periode, een grotere kans op overleving en mogelijk minder irreversibele longschade.

.

13.1 Potential issues of concern

Kans is zeer gering dat zich een thyreotoxicose voor doet. Mocht dit toch het geval zijn dan zijn klachten van tijdelijke en voorbijgaande aard.

a. Level of knowledge about mechanism of action

Het mechanisme van een thyreotoxicose kan een hyperthyreoïdie veroorzaken door een autonome ontregeling van de schildklier. Maar kan ook tijdelijk een hypothyreoïdie veroorzaakt worden door blokkade van Jodium opname door de schildklier.

b. Previous exposure of human beings with the test product(s) and/or products with a similar biological mechanism

In eerdere studies worden bij de door ons gebruikte dosering en toedieningsfrequentie geen relevante bijwerkingen gemeld **[10,11,12]**

c. Can the primary or secondary mechanism be induced in animals and/or in *ex-vivo* human cell material?
Niet bekend

d. Selectivity of the mechanism to target tissue in animals and/or human beings
Niet bekend

e. Analysis of potential effect
Er zijn meerdere in vitro studies en diermodel studies waarbij inactivatie van virussen door jodium is aangetoond.

f. Pharmacokinetic considerations
Jodium wordt door de darmen snel opgenomen in het bloed. Jodium wordt actief in de schildklier ogenomen en daarnaast relatief snel uitgescheiden in meerdere weefsels zoals slijmvliezen.

g. Study population
Alle patienten van 18 jaar en ouder, die worden opgenomen in het ziekenhuis en positief getest zijn op SARS-CoV-2 infectie worden geworven voor dit onderzoek. Patienten moeten wilsbekwaam zijn en in staat om de patienten informatiebrief te lezen en het informed consent formulier te ondertekenen.

h. Interaction with other products
Niet van toepassing

i. Predictability of effect
De verwachting is dat jodium een gunstig effect zal hebben op het beloop van de COVID-19 ziekte

j. Can effects be managed?
Mocht in het zeer zeldzame geval van een ontregeling van de schildklier optreden dan is dit doorgaans van tijdelijke en voorbijgaande aard. Mocht niet het geval zijn dan zijn er medicijnen waarmee je deze ontregeling goed zou kunnen behandelen

13.2 Synthesis
De kans op medisch relevante bijwerkingen is zeer beperkt in de door ons gekozen dosering en toedieningsfrequentie. Mocht dit onverhoopt toch gebeuren dan zijn deze van voorbijgaande aard of anders goed met medicijnen te behandelen.

14. REFERENCES

1. Kelly F.C. (1961). **Iodine in Medicine and Pharmacy since its Discovery 1811-1961**. *Proceedings of the Royal Society of Medicine*, *54*(October) 831-836 https://doi.org/10.1177/003591576105401001

2. Derscheid, R. J., Van Geelen, A., Berkebile, A. R., Gallup, J. M., Hostetter, S. J., Banfi, B., McCray, P. B., & Ackermann, M. R. (2014). **Increased concentration of iodide in airway secretions is associated with reduced respiratory syncytial virus disease severity**. *American Journal of Respiratory Cell and Molecular Biology*, *50*(2), 389–397. https://doi.org/10.1165/rcmb.2012-0529OC

3. Fischer, A. J., Lennemann, N. J., Krishnamurthy, S., Pócza, P., Durairaj, L., Launspach, J. L., Rhein, B. A., Wohlford-Lenane, C., Lorentzen, D., Bánfi, B., & McCray, P. B. (2011). **Enhancement of respiratory mucosal antiviral defenses by the oxidation of iodide**. *American Journal of Respiratory Cell and Molecular Biology*, *45*(4), 874–881. https://doi.org/10.1165/rcmb.2010-0329OC

4. Blum, S. (1914). **Iodine a specific germicide in respiratory affections**: Preliminary Report. In *California state journal of medicine* Vol. 12, Issue 5, pp. 207–208. https://www.ncbi.nlm.nih.gov/pmc/articles/PMC1641102/

5. Menon, I. (1959). **The 1957 pandemic of influenza in India**. *Bulletin of the World Health Organization*. 20,199-224 https://www.ncbi.nlm.nih.gov/pmc/articles/PMC2537734/

6. World Health Organization. **Coronavirus disease 2019. 2020.** https://doi.org/10.1001/jama.2020.2633

7. Delange, F., De Benoist, B., Bürgi, H., Azizi, F., Hajipour, R., Benmiloud, M., Chen, Z. P., Dussault, J., Foo, L. C., Djokomoeljanto, R., Hartono, B., Hollowell, J. G., Irie, M., Jooste, P., Laurberg, P., Lozanov, B., Pretell, E., Rendl, J., Sinawat, S., … Zimmermann, M. (2002). **Determining median urinary iodine concentration that indicates adequate iodine intake at population level**. *Bulletin of the World Health Organization*, *80*(8), 633–636 https://doi.org/10.1590/S0042-96862002000800007

8. Olivieri, A., Di Cosmo, C., De Angelis, S., Da Cas, R., Stacchini, P., Pastorelli, A., & Vitti, P. (2017). **The way forward in Italy for iodine**. In *Minerva Medica*. Apr;108 (2):159-168 https://doi.org/10.23736/S0026-4806.17.04877-7

9. Olivieri, A., Tonacchera, M., & Vitti, P. (2012). **Summary of the first report on the iodine nutritional status in Italy**. *Iodine Global Netword Organization*, p 2–4. https://www.ign.org/cm_data/Summary_of_the_report_on_iodine_nutritional_status_in_Italy.pdf

10. Furnée, C.A. (1997). **Prevention and Control of Iodine Deficiency: a review of a study on the effectiveness of oral iodized oil in Malai.** European Journal of Clinical Nutrition S9-S10 https://pubmed.ncbi.nlm.nih.gov/9598786/

11. Kimball, O., & Marine, D. (1918). **The prevention of simple goiter in man**. Arch Int med, july;41-44 https://jamanetwork.com/journals/jamainternalmedicine/article-abstract/654242

12. Leverge, R., Bergmann, J. F., Simoneau, G., Tillet, Y., & Bonnemain, B. (2003). **Bioavailability of oral vs intramuscular iodinated oil (Lipiodol UF) in healthy subjects**. *Journal of Endocrinological Investigation.* 26(2 Suppl):20-26 https://pubmed.ncbi.nlm.nih.gov/12762636/?from_term=goiter+treatment+iodine+annual

13. Bruger, M., & Member, S. (1943). **On the excretion of iodine in the saliva**. *American Journal of Physiology-Legacy Content.*139 (2): 212-216 https://doi.org/10.1152/ajplegacy

14. Taylor, G. R., & Butler, M. (1982). **A comparison of the virucidal properties of chlorine, chlorine dioxide, bromine chloride and iodine**. *Journal of Hygiene.* (89), 321–328. https://doi.org/10.1017/S0022172400070856

15. McDonnell, G., & Russell, D. (1999). **Antiseptics and disinfectants: activity, action, and resistance.** *Clin. Microbiol. Rev. Jan.: 147–179* https://pubmed.ncbi.nlm.nih.gov/9880479/?from_term=Antiseptics+and+disinfectants%3A+activity%2C+action%2C+and+resistance&from_pos=1

16. Sriwilaijaroen, N., Wilairat, P., Hiramatsu, H., Takahashi, T., Suzuki, T., Ito, M., Ito, Y., Tashiro, M., & Suzuki, Y. (2009). **Mechanisms of the action of povidone-iodine against human and avian influenza A viruses: Its effects on hemagglutination and sialidase activities.** *Virol J.Aug 13; 6:124.* https://doi.org/10.1186/1743-422X-6-124

17. Eggers, M. (2019). **Infectious Disease Management and Control with Povidone Iodine**. *Infectious Diseases and Therapy*. (8):581-593 https://doi.org/10.1007/s40121-019-00260-x

18. Kenzhebekova, R. T., Abekova, A. O., Raziyeva, K. D., Abramova, Z. S., Islamov, R. A., Nersesyan, A. K., & Ilin, A. I. (2018). **Investigation of the impact of iodine coordination compound on production of interleukin-4 and interferon-γ in vitro and primary evaluation of local irritation in vivo**. *Int. J. Biol. Chem. Vol 11, № 2, p.* 4–10. https://doi.org/10.26577/ijbch-2019-1-339

19. Sharma, S., Saimbi, C. S., Koirala, B., & Shukla, R. (2008**). Effect of various mouthwashes on the levels of interleukin-2 and interferon-γ in chronic gingivitis**. *Journal of Clinical Pediatric Dentistry*. 32 (2):111-114 https://doi.org/10.17796/jcpd.32.2.u01p13556116147б

20. Snelgrove, R. J., Edwards, L., Rae, A. J., & Hussell, T.(2006) **An absence of reactive oxygen species improves the resolution of lung influenza infection**. Eur. J. Immunol. 36: 1364–1373 https://doi.org/10.1002/eji.200635977

21. Apostolov, K. (1980).**The effects of iodine on the biological activities of myxoviruses**. *Journal of Hygiëne*. 84, 381-388 https://doi.org/10.1017/S0022172400026905

22. Eggers, M., Koburger-Janssen, T., Eickmann, M., & Zorn, J. (2018). **In Vitro Bactericidal and Virucidal Efficacy of Povidone-Iodine Gargle/Mouthwash Against**

Respiratory and Oral Tract Pathogens. *Infectious Diseases and Therapy*, 7(2), 249–259. https://doi.org/10.1007/s40121-018-0200-7

23. Sattar, S. A., Springthorpe, V. S., Karim, Y., & Loro, P.(1989). **Chemical disinfection of non-porous inanimate surfaces experimentally contaminated with four human pathogenic viruses**. Epidemiology and Infection, 102(3), 493–505. https://doi.org/10.1017/S0950268800030211

24. Winkler, R. (2015). **Iodine—A Potential Antioxidant and the Role of Iodine/Iodide in Health and Disease.** Natural Science, 7, 548-557. https://doi.org/10.4236/ns.2015.712055

25. Shoemake, B. M., Vander Ley, B. L., Newcomer, B. W., & Heller, M. C. (2018). **Efficacy of Oral Administration of Sodium Iodide to Prevent Bovine Respiratory Disease Complex**. Journal of Veterinary Internal Medicine, 32(1), 516–524. https://doi.org/10.1111/jvim

26. Zou, L., **SARS-CoV-2 viral load in upper respiratory specimens of infected patients.** New England Journal of Medicine, (2020) 382(12), 1175–1177. https://doi.org/10.1056/NEJMc2000231

27. Sisk, J. et al. **Coronavirus S protein-induced fusion is blocked prior to hemifusion by Abl kinase inhibitors.** Journal of General Virology 2018; 99: 619–630 https://www.ncbi.nlm.nih.gov/pmc/articles/PMC6537626/pdf/jgv-99-619.pdf

28. Abdel-Mottaleb, M., **In search for effective and safe drugs against SARS-CoV-2: Part II; The role of selected salts and organometallics of copper, zinc, selenium and iodine food supplements.** (mei 5, 2020) https://chemrxiv.org/articles/In_Search_for_Effective_and_Safe_Drugs_Against_SARS-CoV-2_Part_II_the_Role_of_Selected_Salts_and_Organometallics_of_Copper_Zinc_Selenium_and_Iodine_Food_Supplements/12234743/1

Bijlage 1.

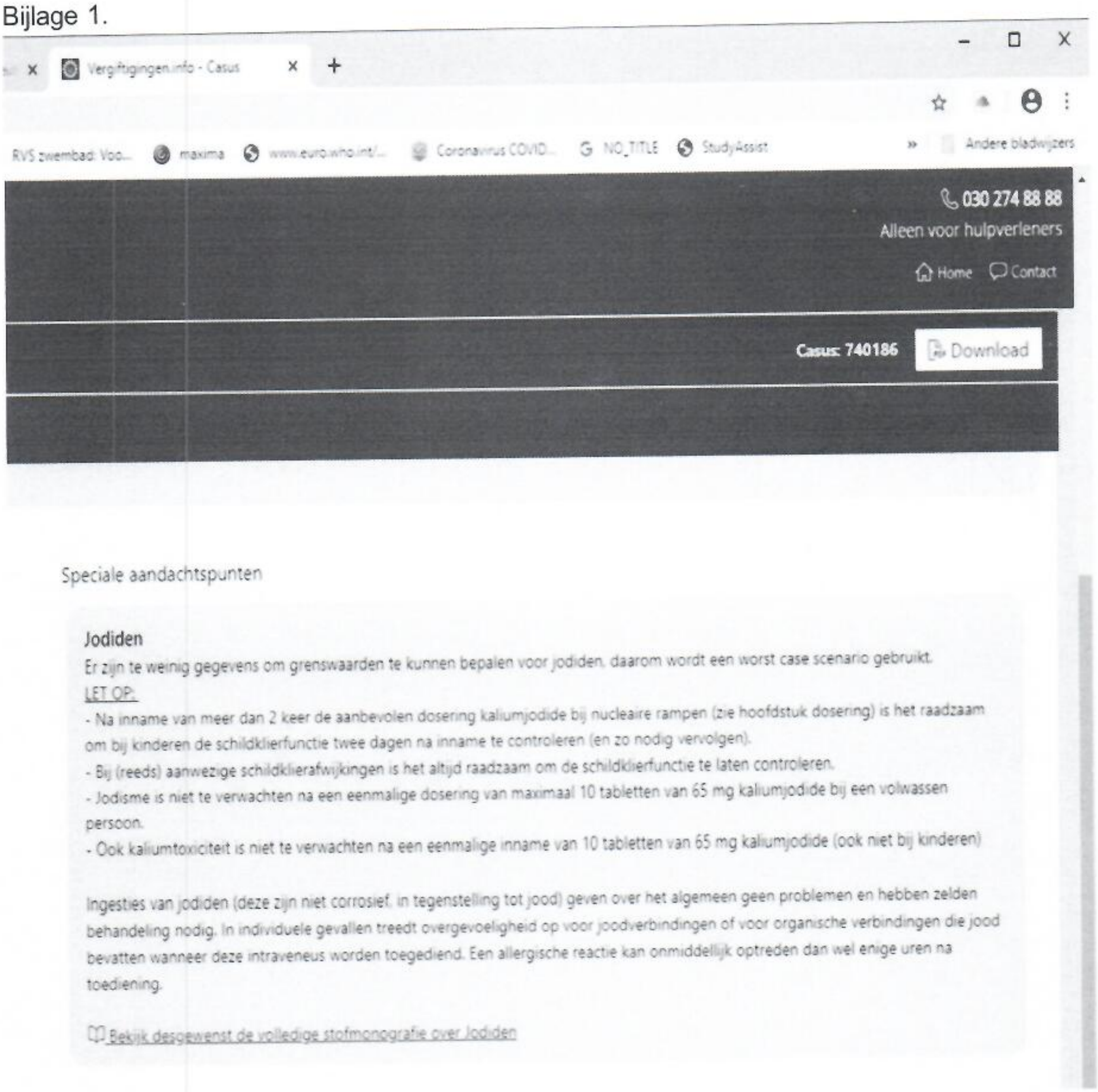
Vergiftigingen.info - Casus

RVS zwembad: Voo... maxima www.euro.who.int/... Coronavirus COVID... NO_TITLE StudyAssist Andere bladwijzers

030 274 88 88
Alleen voor hulpverleners
Home Contact

Casus: 740186 Download

Speciale aandachtspunten

Jodiden

Er zijn te weinig gegevens om grenswaarden te kunnen bepalen voor jodiden, daarom wordt een worst case scenario gebruikt.

<u>LET OP:</u>

- Na inname van meer dan 2 keer de aanbevolen dosering kaliumjodide bij nucleaire rampen (zie hoofdstuk dosering) is het raadzaam om bij kinderen de schildklierfunctie twee dagen na inname te controleren (en zo nodig vervolgen).
- Bij (reeds) aanwezige schildklierafwijkingen is het altijd raadzaam om de schildklierfunctie te laten controleren.
- Jodisme is niet te verwachten na een eenmalige dosering van maximaal 10 tabletten van 65 mg kaliumjodide bij een volwassen persoon.
- Ook kaliumtoxiciteit is niet te verwachten na een eenmalige inname van 10 tabletten van 65 mg kaliumjodide (ook niet bij kinderen)

Ingesties van jodiden (deze zijn niet corrosief, in tegenstelling tot jood) geven over het algemeen geen problemen en hebben zelden behandeling nodig. In individuele gevallen treedt overgevoeligheid op voor joodverbindingen of voor organische verbindingen die jood bevatten wanneer deze intraveneus worden toegediend. Een allergische reactie kan onmiddellijk optreden dan wel enige uren na toediening.

<u>Bekijk desgewenst de volledige stofmonografie over Jodiden</u>
